# Semi-Supervised 2D Pose Estimation for Underwater Divers via Limb-Heatmap-Guided Bidirectional Cross-Representation Consistency Learning

**DOI:** 10.3390/s26216833

**Published:** 2026-10-29

**Authors:** Kaidong Wang, Yi Yang, Qingbo Wei, Xingqun Zhou, Zhiqiang Hu

**Affiliations:** 1 State Key Laboratory of Robotics and Intelligent Systems, Shenyang Institute of Automation, Chinese Academy of Sciences, Shenyang 110016, China; wangkaidong@sia.cn (K.W.);; 2 University of Chinese Academy of Sciences, Beijing 100049, China; 3 School of Electronic and Information Engineering, Harbin Institute of Technology, Shenzhen 518055, China

**Keywords:** underwater visual perception, diver pose estimation, semi-supervised learning, cross-representation consistency, limb heatmaps

## Abstract

Underwater image degradation and occlusion make diver keypoints less visually discernible, leading to substantial localization errors in the keypoint pseudo-labels generated by existing semi-supervised methods. These errors can then accumulate and propagate during consistency learning. Moreover, these methods do not fully exploit the human structural information available in unlabeled images. To address these limitations, a limb-heatmap-guided bidirectional cross-representation consistency method is proposed for semi-supervised learning. A keypoint network and a limb network are employed to learn point-level keypoint responses and region-level limb-structure responses, respectively. For labeled data, limb labels are generated automatically from keypoint annotations and a predefined skeletal topology, without additional annotations. For unlabeled data, intra-representation consistency learning is performed for both networks between the easy-augmentation and hard-augmentation views, and bidirectional cross-representation supervision is applied between the two networks. In the keypoint-to-limb path, keypoint predictions are converted into limb pseudo-labels to supervise the limb network; in the limb-to-keypoint path, limb heatmaps reconstructed from keypoint predictions are compared with the predictions of the limb network to constrain the keypoint configuration. During model inference, only the keypoint network is retained, with no additional inference cost. On the independent test set of a single underwater diver dataset constructed for this study, the proposed method achieves a mean PCKh@0.5 of 80.07% using all labeled training data and outperforms the comparison methods at labeled-data proportions of 25%, 50%, 75%, and 100%. These results demonstrate that region-level limb structure provides effective complementary supervision for point-level keypoint localization within the evaluated setting. Broader applicability requires further validation.

## 1. Introduction

Collaborative operations between underwater robots and divers require highly reliable information exchange. Because of the distinctive characteristics of underwater environments, speech-based interaction and device-based control via remote-control devices or portable terminals, both of which are commonly used for human–robot interaction on land, cannot be applied directly to underwater operations. In this context, 2D pose estimation can localize the body keypoints of divers in underwater images, providing important visual cues for underwater robots to recognize diver behavior, infer interaction intent, support relative human–robot localization, and conduct coordinated operations. Existing studies have applied 2D pose information of divers to various tasks in underwater human–robot collaboration, including identification of multiple divers [1], information exchange [2], diver safety-state detection [3], diver heading prediction and navigation guidance for underwater robots [4], relative localization and autonomous interactive maneuvering [5,6], and diver-assisted cooperative localization of multiple robots [7]. In recent years, deep-learning-based methods such as OpenPose [8], HRNet [9], and Poseur [10] have substantially advanced general human pose estimation but generally rely on large-scale datasets with fine-grained keypoint annotations [11], while color distortion, reduced contrast, scattering-induced blur, and target occlusion in underwater images further increase the difficulty and cost of keypoint annotation. Therefore, semi-supervised learning that fully exploits a small number of labeled samples together with a large number of unlabeled underwater images offers a practical means of improving 2D pose estimation for underwater divers.

Existing semi-supervised 2D pose estimation methods rely primarily on consistency regularization and pseudo-label learning; representative approaches include PoseDual [12], SSPCM [11], and MultiAugs [13]. These methods typically construct an easy-augmentation view and a hard-augmentation view of the same unlabeled image and use the model prediction on the easy view as a pseudo-label to constrain the output on the hard view. Although this paradigm has achieved strong performance on general human pose datasets, its supervisory information is typically confined to the keypoint-heatmap representation space. In underwater scenes, image degradation and occlusion can readily displace response peaks in keypoint heatmaps. Under these conditions, relying solely on point-level heatmap consistency may introduce erroneous supervision from inaccurate pseudo-labels into model updates, causing localization errors to accumulate and propagate throughout training. Moreover, existing methods have not fully exploited the human structural information contained in unlabeled underwater images. Compared with an individual keypoint, a limb region provides broader spatial support and represents the continuous connection between adjacent keypoints, thereby offering a complementary structural constraint when point-level supervision is unstable.

To address these issues, a limb-heatmap-guided bidirectional cross-representation consistency method is proposed for semi-supervised learning. An auxiliary limb network is introduced alongside the keypoint network to model point-level keypoint responses and region-level limb-structure responses, respectively. For labeled data, limb-heatmap labels are generated automatically from keypoint annotations and a predefined skeletal topology, without requiring additional limb-region annotations. For unlabeled data, each network performs intra-representation consistency learning between the easy-augmentation and hard-augmentation views, while bidirectional cross-representation consistency in the keypoint-to-limb and limb-to-keypoint directions provides complementary supervision. By combining intra-representation constraints within each network with bidirectional cross-representation supervision across the two networks, the proposed method reduces its reliance on pseudo-labels generated solely from keypoint heatmaps and more fully exploits the human structural information in unlabeled underwater diver images, thereby improving the accuracy of semi-supervised pose estimation. The empirical evaluation is limited to a single underwater diver dataset under the reported experimental protocol; generalization to other datasets and field conditions requires further validation. The principal contributions are as follows:

(1) A keypoint-limb dual-representation semi-supervised framework is proposed for underwater diver 2D pose estimation. A keypoint network and a limb network are employed to represent point-level keypoint responses and continuous region-level limb structures using keypoint and limb heatmaps, respectively. Incorporating limb-structure representations into learning from unlabeled data addresses the limited modeling and utilization of limb-structure information in existing semi-supervised methods.
(2) A bidirectional cross-representation consistency mechanism between keypoint and limb heatmaps is developed. For each unlabeled sample, the keypoint-to-limb path uses keypoint predictions from the easy-augmentation view to generate confidence-weighted continuous limb pseudo-labels to supervise limb predictions on the hard-augmentation view. Conversely, the limb-to-keypoint path differentiably reconstructs limb heatmaps from keypoint predictions on the hard-augmentation view and constrains the spatial configuration of the keypoints through a region-overlap loss. This mechanism enables bidirectional information transfer between point-level keypoint and region-level limb representations, providing complementary structural supervision for keypoint localization on unlabeled data.
(3) Systematic comparative and ablation experiments were conducted on an underwater diver dataset constructed for this study. Using 100% of the labeled training set, the proposed method achieved a mean PCKh@0.5 of 80.07%, outperforming all compared semi-supervised methods. It achieved the highest accuracy when 25%, 50%, 75%, and 100% of the labeled training data were used. Ablation results supported the effectiveness of each consistency constraint.

## 2. Related Work

### 2.1. 2D Human Pose Estimation

Keypoint heatmaps are the predominant output representation in 2D human pose estimation. In this representation, a model predicts a response map for each keypoint and decodes its coordinates from the response peak. The accuracy of heatmap prediction has been improved through advances in network architecture and feature representation, including multiscale feature fusion and intermediate supervision in the stacked hourglass network [14], a ResNet [15] backbone with deconvolution layers in SimpleBaseline [16], parallel multiresolution representations with repeated information exchange in HRNet [9], and long-range spatial modeling with the Transformer [17] in TransPose [18].

Heatmap-based methods are also susceptible to biases introduced by data transformation and coordinate encoding and decoding. DARK [19] reduces quantization error through unbiased encoding centered at subpixel ground-truth locations and subpixel decoding based on a Taylor expansion of the predicted heatmap distribution. UDP [20] addresses bias in continuous-space data transformations and in coordinate encoding and decoding that combine classification and regression, thereby reducing keypoint-localization bias.

Human pose is determined not only by keypoint locations but also by skeletal connectivity. Cao et al. [8] introduced part affinity fields (PAFs), which encode two-dimensional directional fields between adjacent keypoints for multi-person keypoint association. Sun et al. [21] represented poses as directed child-to-parent bone vectors in a skeletal tree and combined them along skeletal paths to model structural relationships. Zhuang et al. [22] encoded fixed-width regions between connected joints as scalar limb-region maps and fused the predicted maps with image features to guide joint-heatmap regression. Duan et al. [23] proposed PoseConv3D, which encodes 2D poses as joint or limb heatmaps and stacks them along the temporal dimension into a 3D heatmap volume for skeleton-based action recognition. Du et al. [24] proposed AnatPose, which jointly predicts keypoint, bone, and symmetry heatmaps and processes anatomically ordered coarse heatmaps with a bidirectional recurrent neural network to learn dependencies among body parts. Although these methods explicitly exploit skeletal structure, they use structural representations mainly for multi-person keypoint association, structured coordinate regression, fully supervised pose-structure modeling, or skeleton-based action recognition, rather than bidirectional supervision between keypoint and limb heatmaps in semi-supervised pose estimation.

### 2.2. Semi-Supervised 2D Human Pose Estimation

Semi-supervised 2D human pose estimation primarily exploits unlabeled data through consistency regularization and pseudo-label learning. Moskvyak et al. [25] learned pose-invariant semantic keypoint representations while predicting keypoint heatmaps. Their method enforces semantic consistency across corresponding keypoints from different samples. It also imposes transformation equivariance on heatmap predictions and transformation invariance on keypoint representations. Xie et al. [12] addressed the model-collapse problem caused by directly applying consistency regularization to heatmap regression. The stop-gradient prediction on an easy-augmentation view was used as the supervision target for the prediction on the corresponding hard-augmentation view. This strategy was subsequently extended to two independent networks with mutual teaching, resulting in PoseDual. Building on the same easy–hard consistency paradigm, Zhou et al. [13] proposed MultiAugs, which generates multiple hard-augmentation views of the same image and separately constrains each hard-view prediction using the easy-view prediction.

To improve pseudo-label reliability, Wang et al. [26] proposed PLACL, which uses reinforcement learning to search automatically for a sequence of dynamic confidence thresholds suited to different training stages. These thresholds are used to select reliable pseudo-labels, while cross-training mitigates confirmation bias. Huang et al. [11] proposed SSPCM, which introduces an auxiliary teacher network and calculates cross-model position inconsistency using pseudo-labels generated by two teacher networks in the current and previous epochs. The pseudo-label pair with the minimum position inconsistency is selected and fused to obtain a corrected supervision target. Within a dual-student framework, Yu et al. [27] used multi-view augmentations and a threshold-and-refine procedure to construct a pool of denoised pseudo-heatmaps. They then used the estimated cross-student uncertainty to select the more reliable pseudo-heatmaps generated by the two students as learning targets.

Overall, the supervisory information used in existing semi-supervised 2D human pose-estimation methods is largely confined to the keypoint-heatmap representation space and does not explicitly include limb-structure information from unlabeled data. In contrast, the proposed method introduces an auxiliary limb network and establishes bidirectional cross-representation consistency between keypoint and limb heatmaps, thereby enabling point-level keypoint information and region-level limb-structure information to provide complementary supervision.

## 3. Method

### 3.1. Problem Definition

Semi-supervised 2D pose estimation for underwater divers aims to learn a mapping from underwater diver images to 2D keypoint locations using a small number of labeled images and a large number of unlabeled images. Given an input image $I\in {\mathbb{R}}^{W_{0}\times H_{0}\times 3}$, where $W_{0}$ and $H_{0}$ denote the image width and height, respectively, a general pose estimator $f_{\omega }$ extracts features from the input image and predicts keypoint heatmaps, where ω denotes the learnable model parameters. The model outputs J keypoint heatmaps:

(1) $$H=f_{\omega }(I)\in {\mathbb{R}}^{J\times w_{0}\times h_{0}}$$

where the prediction tensor H contains J keypoint channels, each of which describes the response intensity of the corresponding keypoint at different spatial locations; $w_{0}$ and $h_{0}$ denote the heatmap width and height, respectively. During model inference, the response peak in each channel is mapped to the input-image coordinate system to obtain the 2D keypoint coordinates.

In semi-supervised learning, the training data comprise labeled and unlabeled data. The labeled dataset is defined as

(2) $$D_{l}={\left\{\left(I_{i}^{l},H_{i}^{*}\right)\right\}}_{i=1}^{N_{l}}$$

where $I_{i}^{l}$ denotes the i-th labeled underwater diver image, $H_{i}^{*}\in {\mathbb{R}}^{J\times w_{0}\times h_{0}}$ denotes the keypoint heatmaps generated from its ground-truth keypoint coordinates, and $N_{l}$ denotes the number of labeled samples. The unlabeled dataset is defined as

(3) $$D_{u}={\left\{I_{i}^{u}\right\}}_{i=1}^{N_{u}}$$

where $I_{i}^{u}$ denotes the i-th unlabeled underwater diver image, and $N_{u}$ is the number of unlabeled samples. In typical semi-supervised learning settings, $N_{u}>N_{l}$; that is, the number of unlabeled images exceeds the number of labeled images.

For labeled data, the supervised loss for the keypoint network is defined as the mean squared error between the predicted and ground-truth keypoint heatmaps:

(4) $$L_{s}=\frac{1}{N_{l}}\sum_{i=1}^{N_{l}}{\left\|f_{\omega }\left(I_{i}^{l}\right)-H_{i}^{*}\right\|}_{2}^{2}$$

where $L_{s}$ denotes the supervised loss, and ${\left\|\cdot \right\|}_{2}^{2}$ denotes the squared L2 norm. By minimizing this loss, the model reduces the discrepancy between the predicted and ground-truth response distributions, thereby providing a foundational pose representation for subsequent consistency learning based on unlabeled data.

For unlabeled images, mainstream methods construct supervisory signals through consistency learning based on easy-hard augmentation [12]. For the same unlabeled image $I_{i}^{u}$, an easy augmentation $T_{e}$ and a hard augmentation $T_{h}$ are applied separately, yielding an easy-augmentation view $I_{i}^{e}$ and a hard-augmentation view $I_{i}^{h}$:

(5) $$I_{i}^{e}=T_{e}\left(I_{i}^{u}\right),\;I_{i}^{h}=T_{h}\left(I_{i}^{u}\right)$$

The two views undergo different augmentations, but the underlying semantics of the diver pose should remain unchanged. Because the two views may undergo different geometric transformations, their heatmap responses are not inherently aligned in space and therefore cannot be compared directly on a pixelwise basis. To eliminate this spatial misalignment, the prediction on the easy-augmentation view is aligned with the coordinate system of the hard-augmentation view and detached from gradient computation to serve as the pseudo-label target for the hard-augmentation view. The corresponding consistency loss is defined as

(6) $$L_{u}=\frac{1}{N_{u}}\sum_{i=1}^{N_{u}}{\left\|f_{\omega }\left(I_{i}^{h}\right)-\mathrm{sg}\left[W_{e\to h}\left(f_{\omega }\left(I_{i}^{e}\right)\right)\right]\right\|}_{2}^{2}$$

where $L_{u}$ denotes the unsupervised consistency loss, $W_{e\to h}$ denotes the geometric transformation that aligns the prediction on the easy-augmentation view with the coordinate system of the hard-augmentation view, and sg[⋅] denotes the stop-gradient operation. During training, the prediction on the easy-augmentation view provides a relatively stable target signal, whereas the prediction on the hard-augmentation view is optimized, thereby improving the stability of spatial keypoint responses under hard augmentation.

The basic semi-supervised learning objective integrates heatmap supervision for labeled data with the consistency constraint for unlabeled data and is formulated as follows:

(7) $$\;L_{\mathrm{SSL}}=L_{s}+\lambda_{u}L_{u}\;$$

where $\lambda_{u}$ is the weighting coefficient for the unsupervised consistency loss $L_{u}$. This objective constitutes the basic semi-supervised learning paradigm underlying the proposed method.

### 3.2. Overall Framework

**Figure 1:**
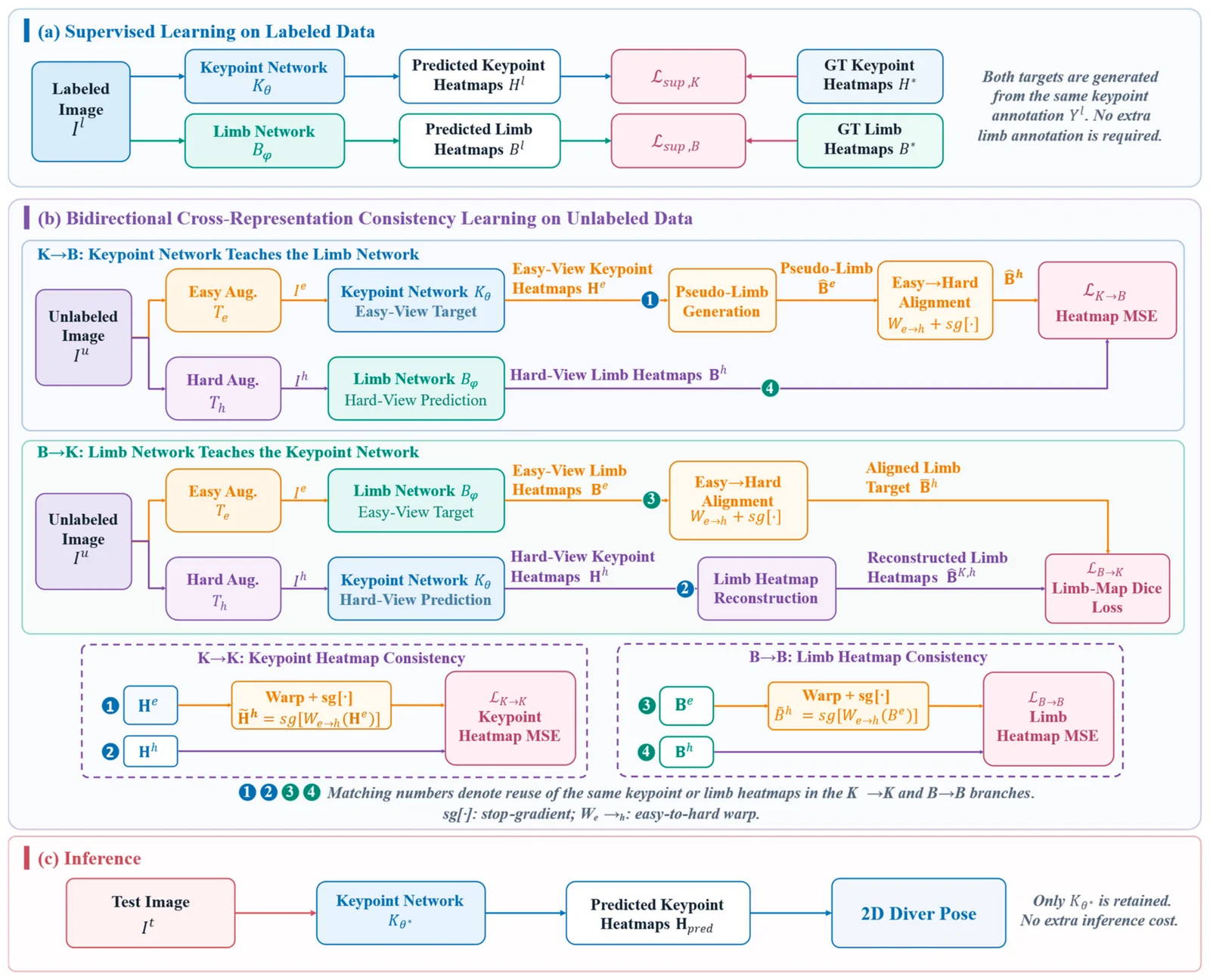
Overall framework of the proposed limb-heatmap-guided bidirectional cross-representation consistency method for semi-supervised learning. (**a**) Supervised learning of the keypoint and limb networks on labeled data; (**b**) intra-network, intra-representation consistency learning and inter-network, bidirectional cross-representation consistency learning on unlabeled data; (**c**) 2D pose inference using only the keypoint network.

Figure 1 illustrates the overall framework of the proposed method, in which a keypoint network $K_{\theta }$ and a limb network $B_{\phi }$ are employed. The two networks have independent architectures and do not share parameters; their learnable parameters are denoted by θ and ϕ, respectively. For an input image I, the outputs of the two networks are expressed as

(8) $$H=K_{\theta }(I)\in {\mathbb{R}}^{J\times w_{0}\times h_{0}},\;B=B_{\phi }(I)\in {\mathbb{R}}^{L\times w_{0}\times h_{0}}$$

where H denotes the keypoint-heatmap tensor containing all keypoint channels, and J is the number of keypoints; B denotes the limb-heatmap tensor containing all limb channels, L denotes the number of valid limb connections in the predefined skeletal topology, and $w_{0}\times h_{0}$ is the spatial resolution of the output heatmaps.

**Figure 2:**
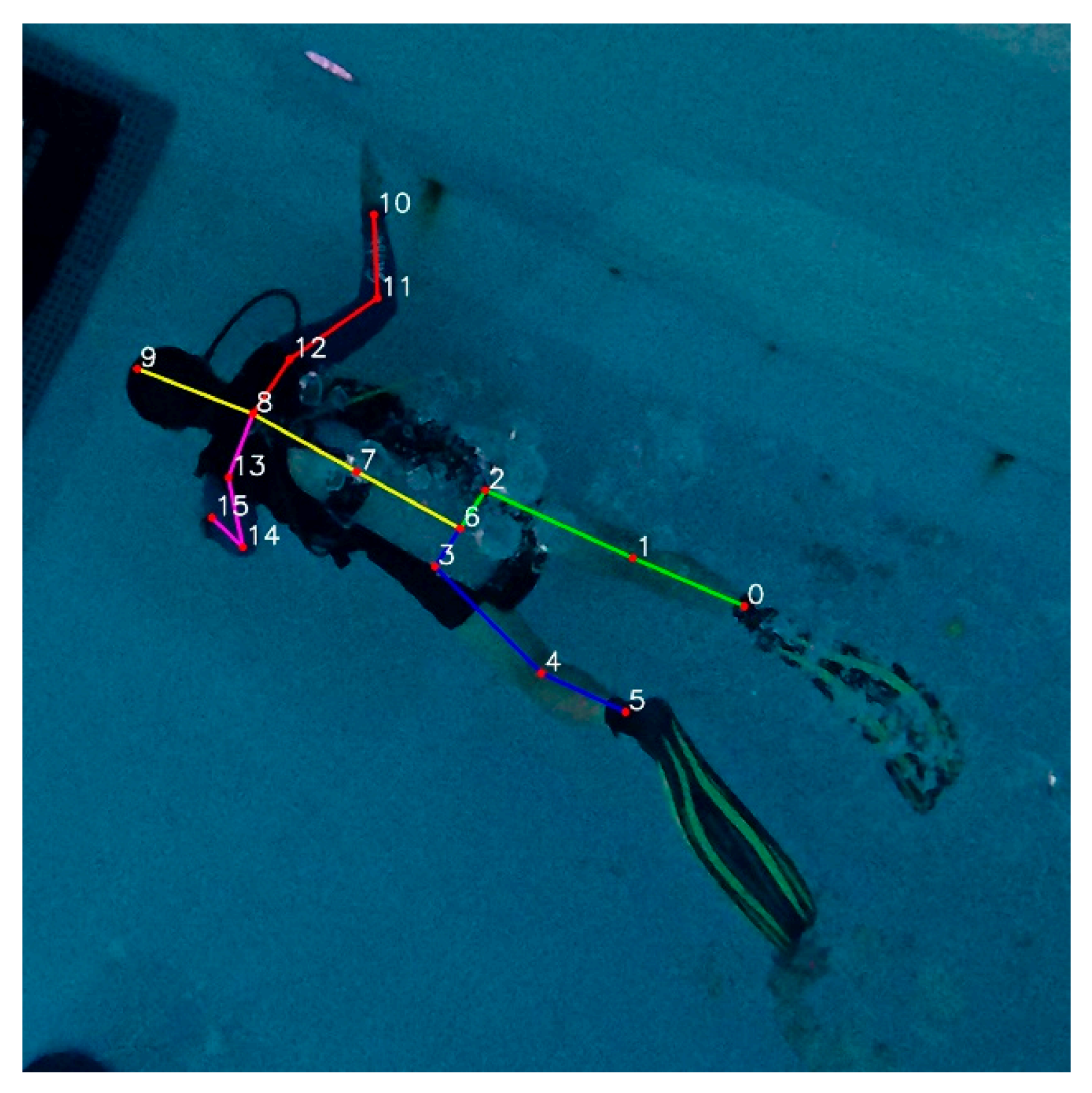
Example and indices of the diver keypoints used in this study. Colored lines indicate skeletal connections and distinguish different body parts; numbers correspond to the keypoint indices listed in Table 1.

To clearly distinguish the heatmap tensors containing all channels from their single-channel heatmaps, the two outputs are further expressed as

(9) $$H={\left\{H_{j}\right\}}_{j=0}^{J-1},\;B={\left\{B_{r}\right\}}_{r=0}^{L-1}$$

where $H_{j}\in {\mathbb{R}}^{w_{0}\times h_{0}}$ denotes the single-channel keypoint heatmap corresponding to keypoint j, and $B_{r}\in {\mathbb{R}}^{w_{0}\times h_{0}}$ denotes the single-channel limb heatmap corresponding to limb r. For heatmap-related notation throughout the paper, boldface letters denote heatmap tensors containing all channels, non-boldface letters with channel indices denote the corresponding single-channel heatmaps, and superscripts are used only to distinguish data views, supervision sources, or generation procedures. The keypoint definitions and indexing scheme shown in Figure 2 and Table 1 are adopted, comprising J=16 keypoints and a corresponding skeletal topology with L=15 valid limb connections. A keypoint heatmap treats an individual keypoint as the basic prediction target and characterizes its local spatial position, whereas a limb heatmap describes the continuous limb-structure region between adjacent keypoints and provides region-level connectivity information. These representations are complementary and form the basis for subsequent supervised learning on labeled data and cross-representation learning on unlabeled data.

For a labeled image $I^{l}$, the keypoint and limb networks use the ground-truth keypoint and limb heatmaps as supervision targets, respectively, enabling the two networks to develop point-level keypoint-localization and region-level limb-structure representation capabilities, respectively. Both supervision targets are generated from the same manually annotated keypoint coordinates, while limb-heatmap generation additionally uses the predefined skeletal topology and therefore introduces no additional manual annotation burden. The generation of the two ground-truth heatmaps and the corresponding supervised losses are described in Section 3.3.

For unlabeled data, the easy-hard augmentation consistency paradigm [12] is adopted. Given an unlabeled image $I^{u}$, an easy augmentation $T_{e}$ generates the easy-augmentation view used to provide a supervisory signal, and a hard augmentation further produces the corresponding hard-augmentation view:

(10) $$I^{e}=T_{e}(I^{u}),\;I^{h}=T_{h}(I^{u})$$

where $I^{e}$ is the easy-augmentation view and $I^{h}$ is the hard-augmentation view. Together, they form an easy-hard-augmentation view pair used to calculate the corresponding consistency losses.

Based on the above augmentation mechanism, four consistency constraints are constructed for each hard-augmentation path. Keypoint-heatmap intra-representation consistency (K→K) and limb-heatmap intra-representation consistency (B→B) constrain the keypoint and limb networks, respectively, to maintain representational consistency between the easy-augmentation and hard-augmentation views. Keypoint-to-limb cross-representation consistency (K→B) converts keypoint predictions on the easy-augmentation view into confidence-weighted limb-heatmap pseudo-labels to supervise limb predictions on the hard-augmentation view. Limb-to-keypoint cross-representation consistency (B→K) uses limb responses on the easy-augmentation view as structural targets, differentiably reconstructs limb heatmaps from keypoint predictions on the hard-augmentation view, and updates the keypoint network using a region-overlap loss between the reconstructed heatmaps and the structural targets. Together, the four constraints provide intra-network, intra-representation supervision and bidirectional cross-representation supervision. Their target-generation procedures and loss formulations are detailed in Section 3.4.

### 3.3. Supervised Learning on Labeled Data

As shown in Figure 1a, under the dual-network, dual-representation supervision framework described in Section 3.2, keypoint- and limb-heatmap labels are generated from the keypoint annotations of labeled samples to supervise the keypoint and limb networks, respectively. Within the labeled dataset $D_{l}$, for a sampled image $I^{l}$, the outputs of the two networks are expressed as

(11) $$H^{l}=K_{\theta }(I^{l})\in {\mathbb{R}}^{J\times w_{0}\times h_{0}},\;B^{l}=B_{\phi }(I^{l})\in {\mathbb{R}}^{L\times w_{0}\times h_{0}}$$

where $H^{l}$ and $B^{l}$ denote the predicted keypoint and limb heatmaps, respectively; J, L, $w_{0}$, and $h_{0}$ have the same meanings as the corresponding symbols in Equation (8). Labels for the keypoint network are generated from keypoint annotations, whereas labels for the limb network are constructed automatically from the keypoint coordinates and the predefined skeletal topology, without requiring additional limb-region annotations. The annotation for the j-th keypoint is $\left(p_{j},v_{j}\right)$, where $p_{j}=(x_{j},y_{j})$ is its annotated 2D coordinate and $v_{j}\in \left\{0,1\right\}$ is its validity indicator. In the heatmap grid space Ω, for any location q, the ground-truth heatmap of this keypoint is generated using a two-dimensional Gaussian distribution centered at $p_{j}$:

(12) $$H_{j}^{*}(q)=\exp\left(-\frac{{\left\|q-p_{j}\right\|}_{2}^{2}}{2\sigma_{k}^{2}}\right),\;\forall q\in \Omega$$

where $\sigma_{k}$ is the scale parameter that controls the spatial extent of the keypoint-heatmap distribution. The heatmap channel corresponding to an invalid keypoint ($v_{j}=0$) is excluded from the supervised-loss calculation.

To construct the limb-heatmap labels, valid limb connections are first identified according to the human skeletal topology adopted in this study. Let ρ(j) denote the parent of the j-th keypoint; the set of valid skeletal connections is then expressed as

(13) $$E=\left\{\left(j,\rho (j))|j\in \{0,\ldots ,J-1\},\;j\neq \rho (j\right)\right\}={\left\{e_{r}=(s_{r},t_{r})\right\}}_{r=0}^{L-1}$$

where $e_{r}$ denotes the r-th limb, with $s_{r}$ and $t_{r}$ denoting the indices of its two endpoint keypoints, respectively. Because the parent of the root keypoint is the root itself, the resulting self-connection does not constitute a valid limb and is excluded from E. For limb $e_{r}=(s_{r},t_{r})$, the coordinates of its two endpoints are $p_{s_{r}}$ and $p_{t_{r}}$, respectively, and the line segment connecting them is denoted by $\bar{p_{s_{r}}p_{t_{r}}}$. The shortest distance from location q in the heatmap to this line segment is expressed as

(14) $$d_{r}(q)=d\left(q,\bar{p_{s_{r}}p_{t_{r}}}\right)$$

**Figure 3:**
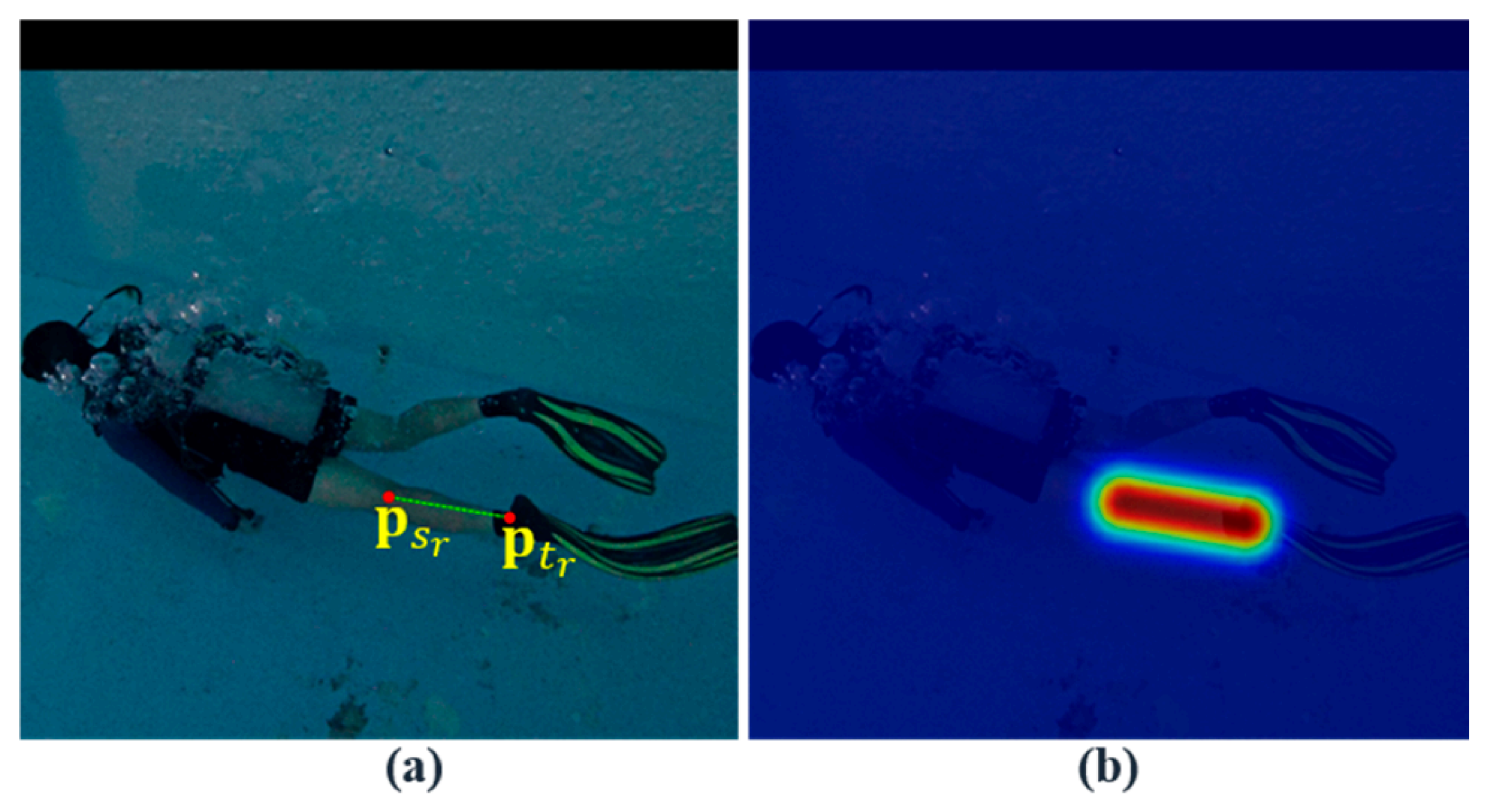
Example of constructing a limb-heatmap label from the coordinates of two endpoint keypoints. (**a**) Locations of the two endpoint keypoints of the r-th limb; (**b**) limb-heatmap label for the r-th limb generated from the two endpoint locations.

Following the method adopted by Duan et al. [23] in PoseConv3D to construct limb heatmaps from adjacent keypoint coordinates, the ground-truth heatmap label for limb r is generated as

(15) $$B_{r}^{*}(q)=\exp\left(-\frac{d_{r}^{2}(q)}{2\sigma_{b}^{2}}\right),\;\forall q\in \Omega$$

where $\sigma_{b}$ controls the width of the limb heatmap, and q denotes any location in the limb-heatmap grid space Ω. Figure 3 shows the heatmap label constructed from the two endpoint coordinates $p_{s_{r}}$ and $p_{t_{r}}$ for the r-th limb. The resulting response distribution is centered on the skeletal line segment connecting the two endpoints and decays smoothly with distance on either side, thereby encoding the connection between adjacent keypoints as a continuous region-level structural label. The limb heatmaps serve a different purpose here than in PoseConv3D [23]. In PoseConv3D, they are used as input representations for skeleton-based action recognition; in the proposed method, they serve as region-level supervision targets for the limb network and are further used for keypoint-limb cross-representation consistency learning on unlabeled data.

A limb contributes to supervision only when both endpoint keypoints have valid annotations. Accordingly, the validity weight of the r-th limb is defined as $\mu_{r}=v_{s_{r}}v_{t_{r}}$. If either endpoint is invalid, $\mu_{r}=0$, and the corresponding limb channel is masked during loss calculation. Based on the predicted and ground-truth keypoint heatmaps, the supervised keypoint loss on labeled data is defined as

(16) $$L_{\sup,K}=\frac{1}{J}\sum_{j=0}^{J-1}v_{j}{\left\|H_{j}^{l}-H_{j}^{*}\right\|}_{2}^{2}$$

where $H_{j}^{l}$ denotes the single-channel heatmap in $H^{l}$ predicted for the j-th keypoint. By minimizing the discrepancy between the predicted response for each keypoint and its corresponding ground-truth heatmap, this loss enables the keypoint network $K_{\theta }$ to learn point-level keypoint localization. Correspondingly, the supervised loss for the limb network is defined as

(17) $$L_{\sup,B}=\frac{1}{L}\sum_{r=0}^{L-1}\mu_{r}{\left\|B_{r}^{l}-B_{r}^{*}\right\|}_{2}^{2}$$

where $B_{r}^{l}$ denotes the single-channel heatmap in $B^{l}$ predicted for the r-th limb. This loss constrains the limb network to regress continuous region responses consistent with human skeletal connectivity, thereby enabling the limb network to develop the ability to predict limb structures at the region level. The overall supervised loss on labeled data is further expressed as

(18) $$L_{\sup}=\lambda_{\sup,K}L_{\sup,K}+\lambda_{\sup,B}L_{\sup,B}$$

where $\lambda_{\sup,K}$ and $\lambda_{\sup,B}$ are weighting coefficients that balance the supervised keypoint and limb losses.

### 3.4. Bidirectional Cross-Representation Consistency Learning on Unlabeled Data

For unlabeled data, four consistency constraints are constructed on each hard-augmentation path using the easy-hard augmentation mechanism described in Section 3.2; the overall structure is shown in Figure 1b. Specifically, keypoint-heatmap and limb-heatmap intra-representation consistency constrain the keypoint and limb networks, respectively, to produce consistent predictions on the easy-augmentation and hard-augmentation views. Keypoint-to-limb and limb-to-keypoint cross-representation consistency establish bidirectional supervision between point-level keypoint and region-level limb representations, enabling complementary information transfer. The target construction and loss function for each consistency constraint are described below.

**(1) Keypoint-heatmap intra-representation consistency supervision (**K→K**).** As shown in the dashed box at the lower left of Figure 1b, along the K→K keypoint-heatmap consistency path, the keypoint heatmaps predicted on the easy-augmentation view undergo spatial alignment and stop-gradient processing to form pseudo-labels that supervise the keypoint-heatmap predictions on the hard-augmentation view. A keypoint-heatmap consistency constraint across augmented views is established by minimizing the discrepancy between these pseudo-labels and the predictions on the hard-augmentation view in the corresponding keypoint channels. Specifically, the easy-augmentation view $I^{e}$ and hard-augmentation view $I^{h}$ are separately fed into the same keypoint network $K_{\theta }$, yielding the corresponding keypoint heatmaps $H^{e}$ and $H^{h}$:

(19) $$H^{e}\;=K_{\theta }(I^{e})={\left\{H_{j}^{e}\right\}}_{j=0}^{J-1}$$

(20) $$H^{h}=K_{\theta }(I^{h})={\left\{H_{j}^{h}\right\}}_{j=0}^{J-1}$$

where J is the number of keypoints, with $H_{j}^{e}$ and $H_{j}^{h}$ denoting the single-channel heatmaps for the j-th keypoint in the easy-augmentation and hard-augmentation views, respectively. The prediction on the easy-augmentation view is spatially aligned to obtain the keypoint-heatmap pseudo-label ${\tilde{H}}^{h}$ used to supervise the keypoint output on the hard-augmentation view:

(21) $${\tilde{H}}^{h}=\mathrm{sg}[(W_{e\to h}(H^{e})]={\left\{{\tilde{H}}_{j}^{h}\right\}}_{j=0}^{J-1}$$

where sg[⋅] denotes the stop-gradient operator, and $W_{e\to h}(\cdot )$ is a spatial transformation that maps the prediction on the easy-augmentation view to the heatmap coordinate system of the hard-augmentation view according to the geometric-transformation parameters recorded for the two augmentation paths. The aligned target and prediction on the hard-augmentation view therefore have consistent spatial relationships. During backpropagation, the pseudo-label is treated as a fixed supervision target, and no gradient is propagated through it. The gradient of the loss is backpropagated only through the hard-view prediction to update the parameters of the keypoint network. The corresponding consistency loss is

(22) $$L_{K\to K}=\frac{1}{J}\sum_{j=0}^{J-1}{\left\|H_{j}^{h}-{\tilde{H}}_{j}^{h}\right\|}_{2}^{2}\;$$

This loss promotes stable keypoint representations across the easy-augmentation and hard-augmentation views. However, because it constrains only keypoint predictions within the same representation, it cannot explicitly model the continuous structure between adjacent keypoints.

**(2) Limb-heatmap intra-representation consistency supervision (**B→B**).** For each unlabeled sample, the keypoint-to-limb cross-representation consistency path (K→B) constructs limb-heatmap pseudo-labels from keypoint-network predictions on the easy-augmentation view to constrain limb-network outputs on the hard-augmentation view. However, these pseudo-labels are obtained by converting keypoint predictions according to the skeletal topology, and their reliability is susceptible to both keypoint-localization errors and pseudo-label construction errors. To provide the limb network with a stable source of supervision when these pseudo-labels are unreliable, the B→B limb-heatmap consistency path shown in the dashed box at the lower right of Figure 1b is constructed. This path directly uses limb-network predictions on the easy-augmentation view as pseudo-label targets and imposes a consistency constraint on the corresponding limb predictions on the hard-augmentation view.

For an unlabeled image $I^{u}$, the easy-augmentation view $I^{e}$ is fed into the limb network $B_{\phi }$ to obtain the limb heatmaps on the easy-augmentation view $B^{e}$:

(23) $$B^{e}=B_{\phi }(I^{e})={\left\{B_{r}^{e}\right\}}_{r=0}^{L-1}$$

The hard-augmentation view $I^{h}$ on the hard-augmentation path is fed into the limb network $B_{\phi }$ to obtain the corresponding limb heatmaps on the hard-augmentation view $B^{h}$:

(24) $$B^{h}=B_{\phi }(I^{h})={\left\{B_{r}^{h}\right\}}_{r=0}^{L-1}$$

where $B^{e}$ and $B^{h}$ are limb-heatmap tensors containing all limb channels, whereas $B_{r}^{e}$ and $B_{r}^{h}$ denote the single-channel heatmaps corresponding to the r-th limb in the easy-augmentation and hard-augmentation views, respectively.

To impose a consistency constraint on the limb predictions on the easy-augmentation and hard-augmentation views in a common coordinate system, the spatial transformation $W_{e\to h}(\cdot )$ associated with the current hard-augmentation path maps the limb prediction on the easy-augmentation view to the heatmap coordinate system of the hard-augmentation view; a supervision target is then constructed by applying the stop-gradient operation:

(25) $${\tilde{B}}^{h}=\mathrm{sg}\left[W_{e\to h}\left(B^{e}\right)\right]={\left\{{\tilde{B}}_{r}^{h}\right\}}_{r=0}^{L-1}$$

where $W_{e\to h}(\cdot )$ denotes the spatial transformation from the easy-augmentation view to the hard-augmentation view, sg[⋅] denotes the stop-gradient operation, and ${\tilde{B}}^{h}$ denotes the supervision target obtained by spatially aligning and detaching the limb prediction on the easy-augmentation view. During backpropagation, no gradient is computed for this target, whereas the prediction on the hard-augmentation view $B^{h}$ retains its gradient. Accordingly, the B→B intra-representation consistency loss for the current hard-augmentation path is defined as

(26) $$L_{B\to B}=\frac{1}{L}\sum_{r=0}^{L-1}{\left\|B_{r}^{h}-{\tilde{B}}_{r}^{h}\right\|}_{2}^{2}\;$$

where r denotes the limb index, and ${\left\|\cdot \right\|}_{2}^{2}$ is the squared L2 norm. By constraining the response distributions of the same limb channel to remain consistent across the easy-augmentation and hard-augmentation views, this loss improves the stability of continuous limb-structure predictions under different image augmentations. Unlike the cross-representation supervision provided by the K→B path, the B→B path conveys supervisory information directly within the limb-heatmap space, without converting keypoint predictions into limb-heatmap pseudo-labels. Thus, the two paths constrain limb predictions on the hard-augmentation view from the complementary perspectives of cross-representation structural association and intra-representation cross-view stability.

**Figure 4:**
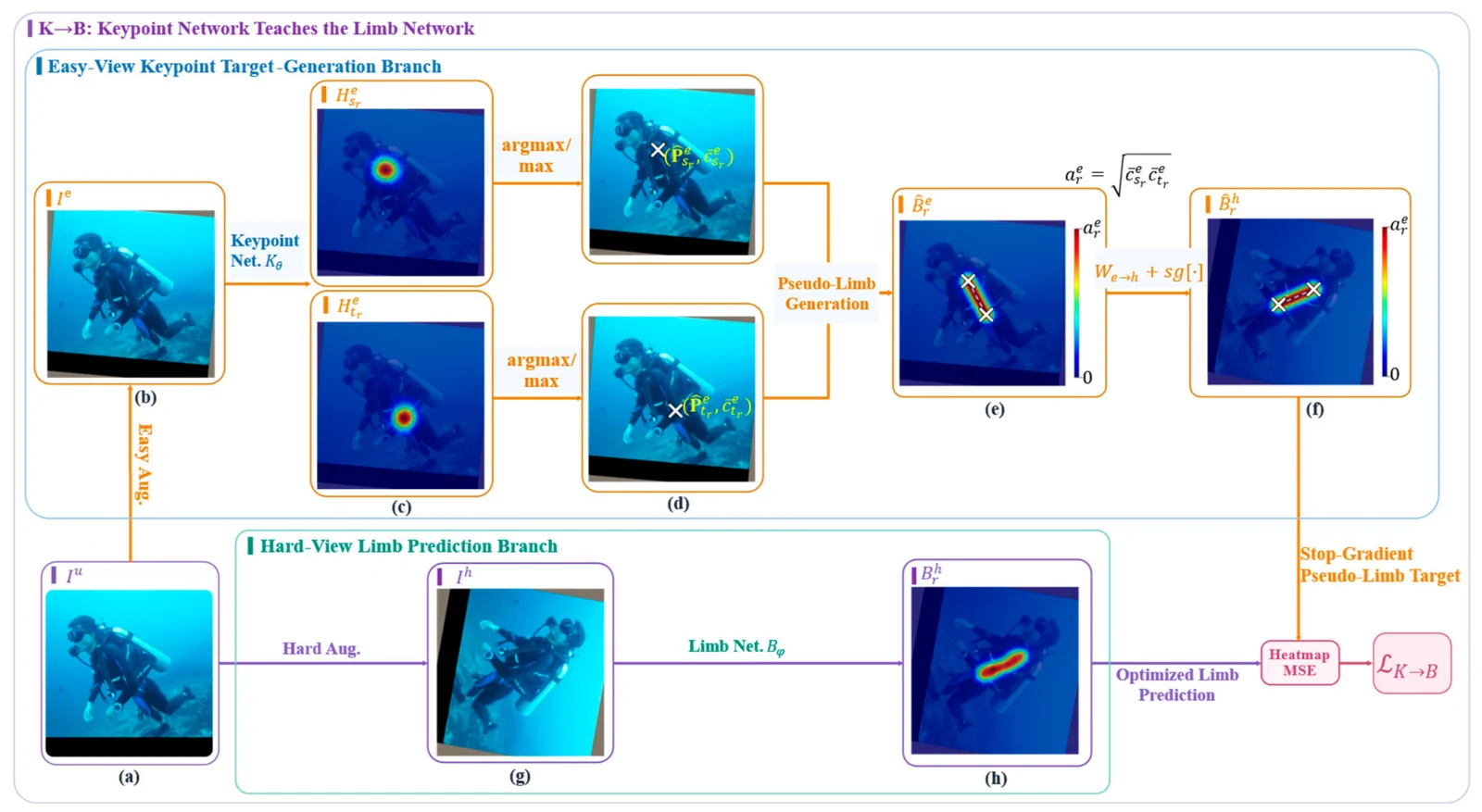
Confidence-weighted keypoint-to-limb (K→B) cross-representation consistency. Using the r-th limb in the easy view as an example, the figure illustrates how predictions for its $s_{r}$-th and $t_{r}$-th endpoint keypoints are used to generate a confidence-weighted limb-heatmap pseudo-label that supervises the corresponding limb prediction on the hard view. (**a**) Unlabeled image; (**b**) easy view; (**c**) heatmaps of the two endpoint keypoints; (**d**) decoded endpoint coordinates and bounded confidence scores; (**e**) confidence-weighted limb-heatmap pseudo-label; (**f**) limb-heatmap pseudo-label after spatial alignment and stop-gradient processing; (**g**) hard view; (**h**) limb-network prediction on the hard view.

**(3) Confidence-weighted keypoint-to-limb cross-representation consistency supervision (**K→B**).** Figure 4 illustrates the construction and use of pseudo-labels for keypoint-to-limb cross-representation consistency. This path uses keypoint-network predictions on the easy-augmentation view to generate limb-heatmap pseudo-labels according to the predefined skeletal topology. After spatial alignment, these pseudo-labels supervise limb-network predictions on the hard-augmentation view. For each limb, the response amplitude of its pseudo-label is jointly determined by the prediction confidence of the two endpoint keypoints, thereby reducing the influence of low-confidence keypoints on cross-representation supervision.

According to Equation (19), for the easy view $I^{e}$, the keypoint network outputs the keypoint-heatmap tensor $H^{e}$. The single-channel heatmap in $H^{e}$ corresponding to the j-th keypoint is denoted by $H_{j}^{e}$. The predicted coordinate of the j-th keypoint is determined by the location of the maximum response in $H_{j}^{e}$:

(27) $${\hat{p}}_{j}^{e}\;=\underset{q\in \Omega }{\arg\max}H_{j}^{e}(q)$$

where q∈Ω denotes any two-dimensional location in the heatmap grid Ω. The heatmap response at the predicted coordinate ${\hat{p}}_{j}^{e}\;$ is defined as the raw confidence of the j-th keypoint:

(28) $$c_{j}^{e}\;=H_{j}^{e}\left({\hat{p}}_{j}^{e}\right)=\underset{q\in \Omega }{\max}H_{j}^{e}(q)$$

Because a heatmap response output by the network may lie outside [0,1], the raw confidence $c_{j}^{e}$ is clipped to obtain the bounded confidence ${\bar{c}}_{j}^{e}$:

(29) $${\bar{c}}_{j}^{e}\;=\mathrm{clip}\left(c_{j}^{e},0,1\right)=\min\left\{1,\max\left\{0,c_{j}^{e}\right\}\right\},\;j=0,\ldots ,J-1$$

This operation sets responses below 0 to 0 and those above 1 to 1, while leaving responses within [0, 1] unchanged, thereby limiting the influence of anomalous response values on the amplitude of the limb pseudo-label.

In the skeletal topology defined in Equation (13), the two endpoint keypoint indices of the r-th limb are $s_{r}$ and $t_{r}$. Equation (27) yields the predicted coordinates ${\hat{p}}_{s_{r}}^{e}$ and ${\hat{p}}_{t_{r}}^{e}$ of the two endpoints in the easy view, and Equation (29) then yields the corresponding bounded confidence scores ${\bar{c}}_{s_{r}}^{e}$ and ${\bar{c}}_{t_{r}}^{e}$. The geometric mean of the two endpoint confidence scores is used to define the response amplitude of the pseudo-label for the r-th limb:

(30) $$a_{r}^{e}\;=\sqrt{{\bar{c}}_{s_{r}}^{e}{\bar{c}}_{t_{r}}^{e}}$$

Under this definition, the pseudo-label amplitude is jointly constrained by the prediction confidence of both endpoints. Low confidence at either endpoint suppresses $a_{r}^{e}$, thereby reducing the supervisory strength of the corresponding limb pseudo-label. Based on the limb-heatmap construction defined in Equations (14) and (15), the line segment determined by the predicted coordinates ${\hat{p}}_{s_{r}}^{e}$ and ${\hat{p}}_{t_{r}}^{e}$ serves as the center, and $a_{r}^{e}$ modulates the response amplitude, yielding a single-channel pseudo-heatmap for the r-th limb:

(31) $${\hat{B}}_{r}^{e}(q)\;=a_{r}^{e}\exp\left[-\frac{d^{2}\left(q,\bar{{\hat{p}}_{s_{r}}^{e}{\hat{p}}_{t_{r}}^{e}}\right)}{2\sigma_{b}^{2}}\right],\;q\in \Omega$$

where d(⋅) denotes the shortest distance from location q to the line segment determined by the two predicted endpoints, and $\sigma_{b}$ is the scale parameter controlling the limb-heatmap width. The limb-heatmap pseudo-label comprising all L limb channels is denoted by ${\hat{B}}^{e}={\left\{{\hat{B}}_{r}^{e}\right\}}_{r=0}^{L-1}$.

To use ${\hat{B}}^{e}$ as the supervision target for limb prediction on the hard-augmentation view, the spatial transformation $W_{e\to h}(\cdot )$ maps it to the heatmap coordinate system of the hard-augmentation view according to the geometric-transformation parameters recorded for the current hard-augmentation path. The stop-gradient operation is then applied to the transformed result:

(32) $${\hat{B}}^{h}=\mathrm{sg}\left[W_{e\to h}\left({\hat{B}}^{e}\right)\right]={\left\{{\hat{B}}_{r}^{h}\right\}}_{r=0}^{L-1}$$

where ${\hat{B}}^{h}$ denotes the limb-heatmap pseudo-label aligned with the coordinate system of the hard-augmentation view, and ${\hat{B}}_{r}^{h}$ denotes its r-th channel. The stop-gradient operator $\mathrm{sg}\left[\cdot \right]$ prevents the pseudo-label ${\hat{B}}^{h}$ from participating in gradient backpropagation, thereby blocking gradient transmission through ${\hat{B}}^{e}$ to the keypoint prediction on the easy-augmentation view $H^{e}$. Consequently, the K→B consistency loss does not update the keypoint network through the pseudo-label.

To calculate the consistency loss between the aligned pseudo-label ${\hat{B}}^{e}$ and the limb prediction on the hard-augmentation view, the hard-augmentation view $I^{h}$ is fed into the limb network $B_{\phi }$ to obtain the limb-heatmap tensor $B^{h}$, as defined in Equation (24). The single-channel heatmap corresponding to the r-th limb channel in this tensor is denoted by $B_{r}^{h}$. Accordingly, the K→B cross-representation consistency loss is defined as the average, over all limb channels, of the squared L2 norm of the difference between the hard-view limb prediction $B^{h}$ and the aligned limb-heatmap pseudo-label ${\hat{B}}^{e}$:

(33) $$L_{K\to B}=\frac{1}{L}\sum_{r=0}^{L-1}{\left\|B_{r}^{h}-{\hat{B}}_{r}^{h}\right\|}_{2}^{2}$$

This loss constrains the limb prediction on the hard-augmentation view to remain consistent with the confidence-weighted limb pseudo-label, enabling the limb network to learn continuous limb-structure responses consistent with the keypoint predictions from unlabeled samples.

**Figure 5:**
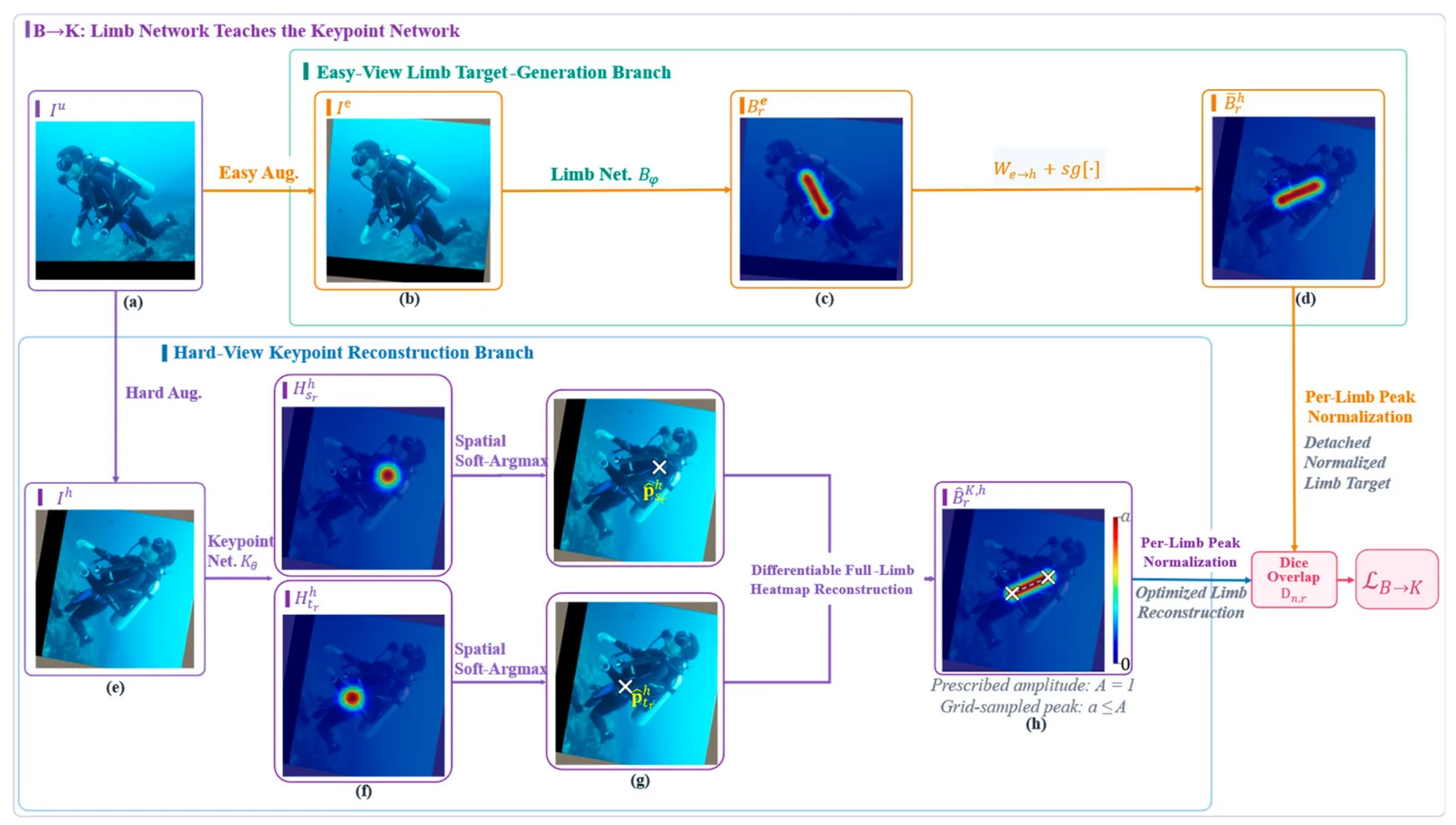
Structure-guided limb-to-keypoint (B→K) cross-representation consistency. Limb r is used as an example to illustrate structural pseudo-label generation and differentiable limb-heatmap reconstruction. (**a**) Unlabeled image; (**b**) easy-augmentation view; (**c**) limb-network heatmap prediction for limb r in the easy-augmentation view; (**d**) structural label map after spatial alignment and stop-gradient processing; (**e**) hard-augmentation view; (**f**) heatmaps of the endpoint keypoints; (**g**) continuous endpoint coordinates decoded using spatial soft-argmax; (**h**) differentiably reconstructed limb heatmap before peak normalization.

**(4) Structure-guided limb-to-keypoint cross-representation consistency supervision (**B→K**).** This path uses limb-network responses on the easy-augmentation view as structural pseudo-labels and differentiably reconstructs the corresponding limb heatmaps from predicted endpoint keypoints in the hard-augmentation view. The outputs of the two networks are thereby mapped into a common, comparable limb-representation space, as shown in Figure 5. Compared with constraining only individual keypoint predictions in the keypoint-heatmap space, the reconstructed limb heatmaps additionally encode the location, orientation, length, and spatial coverage of the connections between adjacent keypoints. Therefore, imposing a region-overlap constraint between the structural pseudo-labels and reconstructed limb heatmaps transfers the dense skeletal structure learned by the limb network to the keypoint network and imposes a topological-consistency constraint on local keypoint predictions.

Specifically, Equation (23) expresses the prediction of the limb network on the easy-augmentation view $I^{e}$ as the limb-heatmap tensor $B^{e}$. The r-th channel of this tensor is the single-channel heatmap $B_{r}^{e}$, which corresponds to the r-th limb. To impose structural supervision on keypoint predictions on the hard-augmentation view, $B^{e}$ is mapped to the heatmap coordinate system of the hard-augmentation view according to the geometric-transformation parameters recorded for the current hard-augmentation path. Response clipping and stop-gradient processing are then applied sequentially to the mapped result, yielding the structural heatmap pseudo-label tensor:

(34) $${\bar{B}}^{h}=\mathrm{sg}\left[\mathrm{clip}\left(W_{e\to h}(B^{e}),0,1\right)\right]={\left\{{\bar{B}}_{r}^{h}\right\}}_{r=0}^{L-1}$$

where ${\bar{B}}^{h}$ denotes the structural heatmap pseudo-label tensor aligned with the heatmap coordinate system of the hard-augmentation view, and its r-th channel is denoted by ${\bar{B}}_{r}^{h}$.

The prediction of the keypoint network on the hard-augmentation view $I^{h}$ is expressed in Equation (19) as the keypoint-heatmap tensor $H^{h}$. Within this tensor, the single-channel heatmap corresponding to keypoint j is $H_{j}^{h}$. To allow the gradient of the region-level structural loss to propagate back to the keypoint heatmaps, spatial soft-argmax is used to convert each keypoint heatmap into a continuous coordinate. For keypoint j with heatmap $H_{j}^{h}$, the normalized spatial probability at location q is

(35) $$\pi_{j}^{h}(q)=\exp\left(\eta H_{j}^{h}(q)\right)/\sum_{z\in \Omega }\exp\left(\eta H_{j}^{h}(z)\right)$$

where z denotes any coordinate in the heatmap grid space Ω, and η is the coefficient controlling the sharpness of the probability distribution; η=50 is used in this study. The continuous predicted coordinate of the j-th keypoint in the hard-augmentation view is therefore

(36) $${\hat{p}}_{j}^{h}=\sum_{q\in \Omega }\pi_{j}^{h}(q)q,\;j=0,\ldots ,J-1$$

This probability-weighted procedure preserves the differentiability of the predicted coordinates with respect to their source heatmaps, allowing the gradient of the subsequent structural loss to be backpropagated through the continuous coordinates to the corresponding keypoint heatmaps.

For the r-th limb, the endpoint keypoint indices are $s_{r}$ and $t_{r}$. According to Equation (36), their continuous predicted coordinates in the hard-augmentation view are ${\hat{p}}_{s_{r}}^{h}$ and ${\hat{p}}_{t_{r}}^{h}$, respectively. To transform the point-level predictions of the keypoint network into the region-level representation space of the limb network, the limb-heatmap construction defined in Equation (15) of Section 3.3 is adopted. A limb heatmap is differentiably reconstructed from the predicted coordinates of the two endpoint keypoints and used as the optimization target of the structural-consistency constraint. The reconstructed limb heatmap is defined as

(37) $${\hat{B}}_{r}^{K,h}(q)=\exp\left(-\frac{{\left(d_{r}^{h}(q)\right)}^{2}}{2\sigma_{b}^{2}}\right),\;d_{r}^{h}(q)=d\left(q,\bar{{\hat{p}}_{s_{r}}^{h}{\hat{p}}_{t_{r}}^{h}}\right)$$

where $d_{r}^{h}$ denotes the shortest distance from location q in the heatmap to the line segment $\bar{{\hat{p}}_{s_{r}}^{h}{\hat{p}}_{t_{r}}^{h}}$. Unlike the K→B path, which adjusts the response amplitude of a limb pseudo-label according to endpoint confidence, the B→K path fixes the amplitude coefficient of the reconstructed limb heatmap at 1. This setting is not intended to assess structural-target reliability from the response amplitude of the keypoint heatmap. Instead, it constructs a geometric limb representation uniquely determined by the predicted keypoint locations, such that the subsequent consistency constraint primarily reflects the limb geometry defined by the keypoint locations and their connectivity. Consequently, the keypoint network cannot weaken the structural constraint by reducing its response amplitude; instead, it must adjust the keypoint locations until the location, orientation, and length of the reconstructed limb gradually align with the region-level structural target provided by the limb network.

Because the response scales of the structural heatmap pseudo-label and the reconstructed limb heatmap may differ, per-limb peak normalization is applied separately to the two heatmaps to reduce the influence of amplitude-scale differences on the subsequent region-overlap measurement:

(38) $${\tilde{B}}_{r}^{B,h}=\frac{{\bar{B}}_{r}^{h}}{\max(\underset{z\in \Omega }{\max}{\bar{B}}_{r}^{h}(z),\varepsilon )}$$

(39) $${\tilde{B}}_{r}^{K,h}=\frac{{\hat{B}}_{r}^{K,h}}{\max(\underset{z\in \Omega }{\max}{\hat{B}}_{r}^{K,h}(z),\varepsilon )}$$

where the superscript B indicates that the heatmap originates from the prediction of the limb network on the easy-augmentation view and is spatially aligned, response-clipped, and detached to form the structural heatmap pseudo-label; the superscript K indicates that the heatmap is obtained through differentiable coordinate decoding and limb-heatmap reconstruction from the prediction of the keypoint network on the hard-augmentation view; and the superscript h indicates that both heatmaps are represented in the heatmap coordinate system of the hard-augmentation view. Per-limb peak normalization enables the subsequent measure to reflect primarily the spatial locations and coverage relationship of the limb regions, rather than differences in raw response amplitude. The Dice coefficient is then used to measure the region overlap between the two single-channel limb heatmaps. For the r-th limb on the current hard-augmentation path, the Dice coefficient is defined as

(40) $$D_{r}=\frac{2\sum_{q\in \Omega }{\tilde{B}}_{r}^{K,h}(q){\tilde{B}}_{r}^{B,h}(q)+\varepsilon }{\sum_{q\in \Omega }{\left({\tilde{B}}_{r}^{K,h}(q)\right)}^{2}+\sum_{q\in \Omega }{\left({\tilde{B}}_{r}^{B,h}(q)\right)}^{2}+\varepsilon }$$

where $D_{r}$ denotes the region overlap for the r-th limb on the hard-augmentation path, r is the limb index, L is the number of limb connections in the predefined skeletal topology, and ε is a small constant that ensures numerical stability. A value of $D_{r}$ closer to 1 indicates greater spatial overlap between the two limb representations. Averaging the structural inconsistency over all limbs on the current hard-augmentation path yields the B→K consistency loss:

(41) $$L_{B\to K}=\frac{1}{L}\sum_{r=0}^{L-1}\left(1-D_{r}\right)$$

This loss imposes a structural constraint on keypoint predictions on the hard-augmentation view by comparing the region overlap between the structural heatmap pseudo-labels and the reconstructed limb heatmaps. When endpoint keypoints are displaced or connected incorrectly, the two heatmaps differ in location, orientation, length, or coverage, thereby reducing their region overlap and increasing the loss. The loss gradient is backpropagated sequentially through differentiable limb-heatmap reconstruction and spatial soft-argmax to the keypoint heatmaps on the hard-augmentation view, whereas the structural heatmap pseudo-labels remain detached as supervision targets. In this manner, the B→K path directly transfers region-level skeletal information to the keypoint network, thereby suppressing implausible keypoint configurations. This region-level constraint primarily characterizes structural relationships among keypoints and cannot replace the direct supervision of individual-keypoint positional consistency provided by the K→K path.

Per-limb peak normalization does not assess pseudo-label reliability: weak, spatially inaccurate limb responses may still impose erroneous structural constraints after normalization, particularly under severe underwater degradation or occlusion. The current B→K path does not explicitly weight or filter structural pseudo-labels based on confidence or uncertainty. This remains a limitation of the structural supervision.

For unlabeled data, the overall consistency loss is the weighted sum of four losses: keypoint-heatmap intra-representation consistency, limb-heatmap intra-representation consistency, keypoint-to-limb cross-representation consistency, and limb-to-keypoint cross-representation consistency:

(42) $$L_{\mathrm{unsup}}=\lambda_{KK}L_{K\to K}+\lambda_{BB}L_{B\to B}+\lambda_{KB}L_{K\to B}+\lambda_{BK}L_{B\to K}$$

where $\lambda_{KK}$, $\lambda_{BB}$, $\lambda_{KB}$, and $\lambda_{BK}$ are the weighting coefficients for the four consistency losses described above, respectively.

### 3.5. Overall Objective and Training Strategy

The overall loss function for the complete training process combines the supervised loss in Equation (18) with the consistency loss in Equation (42) and is defined as

(43) $$L_{\mathrm{total}}(t)=\lambda_{\sup,K}L_{\sup,K}+\lambda_{\sup,B}L_{\sup,B}+\lambda_{KK}L_{K\to K}+\lambda_{BB}L_{B\to B}+g(t)\left(\lambda_{KB}L_{K\to B}+\lambda_{BK}L_{B\to K}\right)$$

where the loss terms and their weights follow the definitions given in Section 3.3 and Section 3.4. The function g(t) controls whether the two cross-representation consistency losses K→B and B→K are enabled at different training stages, whereas the two intra-representation consistency losses K→K and B→B remain active throughout training. The switching function g(t) is defined as

(44) $$g(t)=\left\{\begin{array}{ll} 0, & t<t_{w},\\ 1, & t\geq t_{w}. \end{array}\right.$$

where t denotes the current training epoch, and $t_{w}$ denotes the specified epoch at which the bidirectional cross-representation consistency losses begin to participate in optimization. Before this switch, the overall loss comprises the supervised losses on labeled data and the two intra-representation consistency losses K→K and B→B. Once the training epoch reaches $t_{w}$, the two cross-representation consistency losses K→B and B→K are added simultaneously to the overall loss with preset fixed weights, establishing bidirectional complementary supervision between point-level keypoint and region-level limb representations on unlabeled data.

The gradient-transmission mechanism for each consistency path is described separately in Section 3.4. All supervision targets constructed from predictions on the easy-augmentation view undergo stop-gradient processing. Therefore, loss gradients are propagated only through the hard-view prediction branches to update the parameters of the corresponding supervised networks, preventing gradients from propagating backward through the pseudo-label generation paths.

During training, one batch of labeled data and one batch of unlabeled data are sampled in each iteration. The labeled batch is used to calculate the supervised losses for the keypoint and limb networks. For the unlabeled batch, an easy-augmentation view and a hard-augmentation view are generated for each image, and two intra-representation consistency losses and two cross-representation consistency losses are calculated. These losses are weighted and combined according to Equation (43) to form the overall loss for the current iteration, which is used to update both networks jointly.

During inference, as shown in Figure 1c, only the keypoint network is used; the limb network is not invoked, and neither multi-path augmentation nor any of the four consistency losses is applied. The prediction produced by the retained keypoint network for a test image $I^{\mathrm{test}}$ can be expressed as

(45) $$H_{\mathrm{pred}}=K_{\theta^{*}}\left(I^{\mathrm{test}}\right)$$

where $\theta^{*}$ denotes the keypoint-network parameters obtained through training, $K_{\theta^{*}}$ denotes the keypoint network parameterized by $\theta^{*}$, and $H_{\mathrm{pred}}$ denotes the keypoint heatmaps output by this network. For each keypoint, the predicted 2D coordinate is determined from the location of the maximum response in the corresponding channel of $H_{\mathrm{pred}}$ and mapped to the input-image coordinate system through a coordinate transformation. The predicted coordinates of all keypoints jointly constitute the 2D pose of the underwater diver. Because only the keypoint network is retained during deployment, the auxiliary limb network introduced during training increases neither the parameter count nor the inference computation of the deployed model.

## 4. Experiments

### 4.1. Experimental Details

**Dataset.** An underwater diver dataset was constructed from publicly available online videos. The labeled training, unlabeled training, validation, and test sets contain 2536 labeled images, 4480 unlabeled images, 1105 labeled images, and 726 labeled images, respectively, extracted from 46, 95, 37, and 23 source videos. Frames were sparsely sampled, generally at intervals of approximately 30 frames. Source videos do not overlap across the four subsets, preventing frames from the same video from being assigned to different subsets. Manual inspection identified no repeated divers across subsets. Because the videos were collected from heterogeneous online sources, their original resolutions and acquisition conditions varied, and reliable camera-device information was generally unavailable.

Keypoint annotations for the labeled training, validation, and test sets were produced by a third-party annotation service using unified instructions and reference examples defining the 16 keypoints. The authors manually reviewed all annotations in multiple rounds and corrected errors and inconsistencies before use. Occluded keypoints were annotated when their anatomical locations could be reasonably inferred from visible body structure; completely invisible or out-of-image keypoints were marked as invalid.

**Figure 6:**
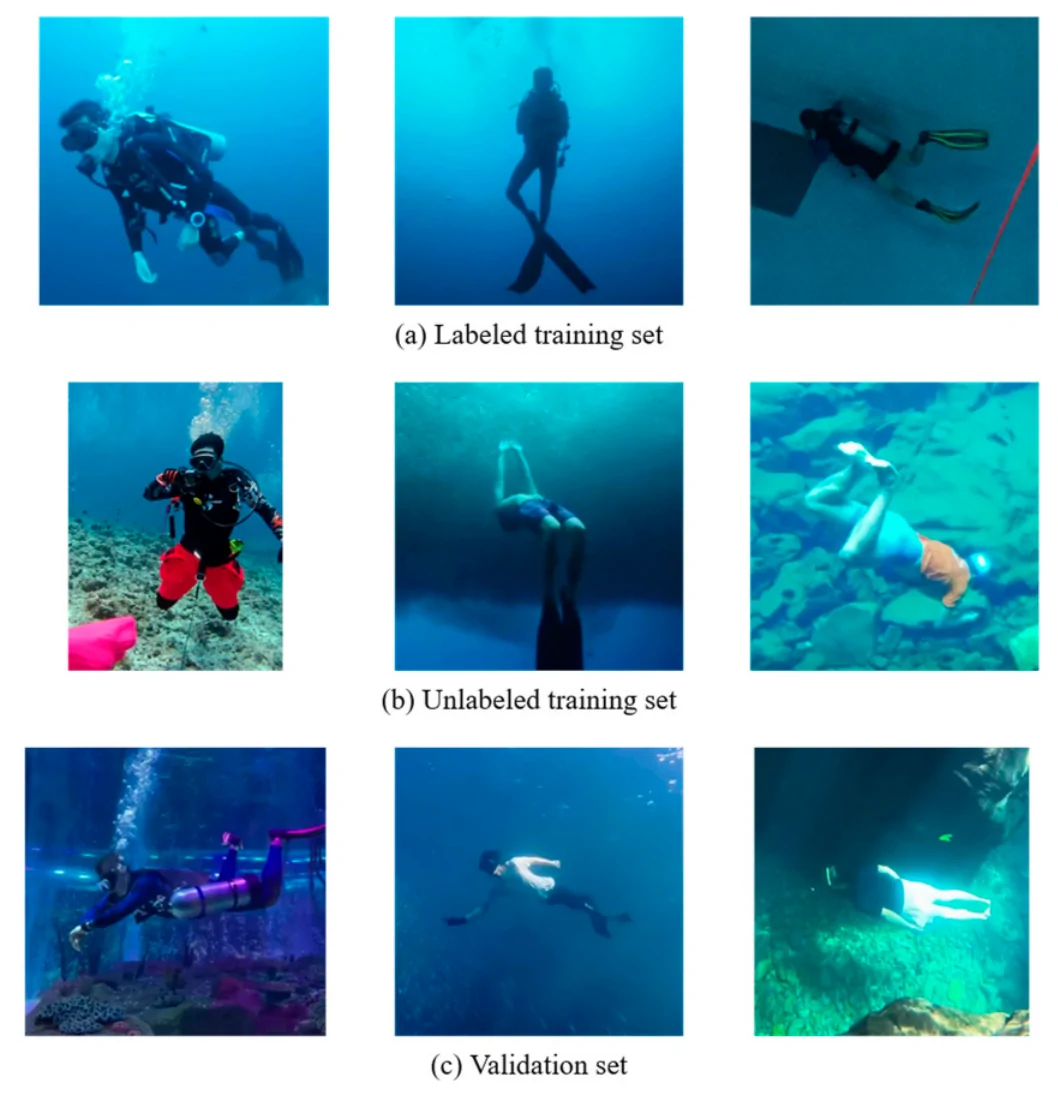
Selected examples from (**a**) the labeled training set, (**b**) the unlabeled training set, and (**c**) the validation set, illustrating variations in aquatic environments, camera viewpoints, breathing equipment, and clothing.

The labeled training set primarily covers ocean, diving-pool, and swimming-pool scenes and mainly contains divers carrying air tanks or other underwater breathing equipment and wearing professional diving suits. The unlabeled training and validation sets cover ponds and various ocean environments, with more diverse camera viewpoints and clothing, more samples exhibiting severe color casts, and more samples of divers without breathing equipment. The test set likewise covers multiple underwater environments and diverse clothing, and includes divers both with and without professional diving equipment. Figure 6 shows selected examples from the labeled training, unlabeled training, and validation sets.

Because manual keypoint annotation of underwater diver images is costly, the labeled training data are relatively limited in both sample size and scene coverage, whereas unlabeled videos are easier to obtain. The unlabeled training set is therefore used to broaden the range of aquatic environments, camera viewpoints, equipment configurations, and clothing appearances available during training. The labeled and unlabeled training sets differ in their scene and appearance coverage, with the latter including variations that are less common in the labeled training set but are also represented in the validation and test sets. These shared scene and appearance characteristics do not imply identical overall data distributions among the unlabeled training, validation, and test sets. The validation set is used for checkpoint selection, whereas the independent test set is reserved for final quantitative evaluation.

**Evaluation metrics.** The percentage of correct keypoints normalized by head size (PCKh) [28] is used to evaluate keypoint-localization performance. PCKh normalizes keypoint-localization errors by a head-reference scale to reduce the influence of body-scale differences. It is calculated as

(46) $$\mathrm{PCKh}@\alpha =\frac{\sum_{i=1}^{N_{\mathrm{test}}}\sum_{j=0}^{J-1}v_{i,j}I\left(\frac{{\left\|{\hat{p}}_{i,j}-p_{i,j}\right\|}_{2}}{l_{i}^{\mathrm{head}}}\leq \alpha \right)}{\sum_{i=1}^{N_{\mathrm{test}}}\sum_{j=0}^{J-1}v_{i,j}}\times 100\%$$

where $N_{\mathrm{test}}$ is the number of test samples, J is the number of keypoints, ${\hat{p}}_{i,j}$ and $p_{i,j}$ denote the predicted and ground-truth coordinates, respectively, of the j-th keypoint in the i-th test sample, $v_{i,j}\in \{0,1\}$ is the validity indicator for that keypoint, and $l_{i}^{\mathrm{head}}$ is the head-reference length of the corresponding test sample. The term ${\left\|{\hat{p}}_{i,j}-p_{i,j}\right\|}_{2}/l_{i}^{\mathrm{head}}$ represents the normalized localization error, where ${\left\|\cdot \right\|}_{2}$ denotes the L2 norm. $I\left(\cdot \right)$ denotes the indicator function, and α is the threshold applied to the normalized localization error. For PCKh evaluation, the head bounding box of each validation and test image was manually annotated as the tightest axis-aligned rectangle enclosing the entire head region. For partially occluded heads, the full head extent was determined with reference to neighboring frames in the original source video. A unified annotation criterion was applied, and the annotations were manually reviewed to maintain consistency. Following the standard evaluation protocol of the MPII Human Pose Dataset [28], the head-reference length $l_{i}^{\mathrm{head}}$ is defined as 0.6 times the diagonal length of the annotated head bounding box. For PCKh@α, a valid keypoint is counted as correct when its normalized localization error does not exceed α. PCKh@0.5 is used as the primary evaluation metric, while PCKh@0.1, PCKh@0.2, PCKh@0.3, and PCKh@0.4 are additionally reported in Table 2b to assess localization accuracy at stricter error tolerances. At a given threshold, a higher PCKh indicates better keypoint-localization performance.

In addition to PCKh, the mean Soft Dice coefficient (mSoftDice) is used to assess the quality of region-level structural predictions from the auxiliary limb network by measuring the overlap between predicted and ground-truth limb heatmaps. For sample i, the Soft Dice coefficient for limb r is defined as

(47) $$D_{i,r}=\frac{2\sum_{q\in \Omega }{\hat{B}}_{i,r}(q)B_{i,r}^{*}(q)+\varepsilon }{\sum_{q\in \Omega }{\left[{\hat{B}}_{i,r}(q)\right]}^{2}+\sum_{q\in \Omega }{\left[B_{i,r}^{*}(q)\right]}^{2}+\varepsilon }$$

where ${\hat{B}}_{i,r}$ and $B_{i,r}^{*}$ denote the predicted and ground-truth heatmaps, respectively, of the r-th limb in the i-th test sample; q denotes a location in the heatmap grid space Ω; and ε is a small constant that ensures numerical stability. Consistent with the definition in Section 3.3, a limb contributes to metric calculation only when both endpoint keypoints have valid annotations. For test sample i, the validity weight of limb r is defined as

(48) $$\mu_{i,r}=v_{i,s_{r}}v_{i,t_{r}}$$

The mSoftDice score on the test set is defined as

(49) $$\mathrm{mSoftDice}=\frac{\sum_{i=1}^{N_{\mathrm{test}}}\sum_{r=0}^{L-1}\mu_{i,r}D_{i,r}}{\sum_{i=1}^{N_{\mathrm{test}}}\sum_{r=0}^{L-1}\mu_{i,r}}$$

where $N_{\mathrm{test}}$ denotes the number of test samples, and L is the number of valid limb connections in the skeletal topology. A higher mSoftDice indicates greater spatial overlap between the predicted and ground-truth limb regions. PCKh evaluates the final localization performance of the keypoint network and serves as the primary evaluation metric in this study. By contrast, mSoftDice is used only as an auxiliary metric in the ablation experiments to analyze how different consistency constraints affect the quality of region-level structural predictions from the auxiliary limb network.

**Network initialization and implementation environment.** HRNet-W48 [9] is used as the backbone for both the keypoint and limb networks in the proposed bidirectional cross-representation consistency method. According to the predefined skeletal topology, the keypoint network outputs 16 keypoint heatmaps, whereas the limb network outputs 15 limb heatmaps. Both networks use an input resolution of 256 × 256 and an output heatmap resolution of 64 × 64. All experiments were conducted on four NVIDIA GeForce RTX 4090 GPUs (NVIDIA Corporation, Santa Clara, CA, USA). A transfer-learning strategy [29] was adopted. The keypoint and limb networks were first pretrained separately on the MPII dataset [28], and the resulting pretrained parameters were then used to initialize the underwater diver keypoint and limb networks. Because the MPII dataset does not include the spine keypoint defined in this study, the midpoint of the line segment connecting the pelvis and thorax keypoints was used as a surrogate annotation for the spine keypoint during MPII pretraining. In the underwater diver dataset constructed for this study, the spine keypoint was manually annotated.

**Optimization strategy and loss parameters.** The Adam optimizer [30] was used, with the labeled- and unlabeled-data batch sizes both set to 12 per GPU. Thus, across four GPUs, the global batches contained 48 labeled and 48 unlabeled samples, respectively. The model was trained for 100 epochs with an initial learning rate of $1\times 10^{-4}$; after epochs 30 and 70, the learning rate was reduced to 0.4 times its preceding value. For each run, the checkpoint achieving the highest validation-set PCKh@0.5 across training epochs was retained. The same selection rule was applied to all methods and experimental settings. The retained checkpoints were evaluated on the independent test set for final quantitative reporting. The scale parameters for the keypoint and limb heatmaps were set to $\sigma_{k}=3$ and $\sigma_{b}=2$, respectively. The weights $\lambda_{\sup,K}$ and $\lambda_{\sup,B}$ of the supervised keypoint and limb losses on labeled data were both set to 1.0. The weights of the consistency losses K→K, B→B, B→K, and K→B on unlabeled data were set to $\lambda_{KK}=1$, $\lambda_{BB}=0.1$, $\lambda_{BK}=0.003$, and $\lambda_{KB}=0.01$, respectively. The first seven epochs served as a warm-up stage for bidirectional cross-representation consistency learning, during which only the supervised losses on labeled data and the intra-representation consistency losses K→K and B→B were optimized. After the warm-up stage, the bidirectional cross-representation consistency losses B→K and K→B were enabled simultaneously. These method-specific hyperparameters were empirically selected through preliminary experiments and were then fixed for all reported experiments.

**Data augmentation.** Affine transformations are commonly used for data augmentation in 2D human pose estimation, introducing geometric perturbations through random scaling and rotation of the input image. Xie et al. [12] denoted an affine transformation with rotation angles sampled from $\left[-30^{{}^{\circ}},30^{{}^{\circ}}\right]$ and scale factors varied within $\left[0.75,1.25\right]$ as A30 and used it as the easy augmentation in easy-hard consistency learning. Given that underwater divers may exhibit arbitrary body orientations, the scale range of A30 was retained, while the rotation-angle sampling interval was expanded from $\left[-30^{{}^{\circ}},30^{{}^{\circ}}\right]$ to $\left[-90^{{}^{\circ}},90^{{}^{\circ}}\right]$.

For each unlabeled image, the easy-augmentation view was generated using the random affine transformation described above. The hard-augmentation view was then generated by sequentially applying Joint Cutout (JC), as used in PoseDual [12], and a second random affine transformation to the easy-augmentation view. The second affine transformation used the same parameter-sampling ranges as the first.

### 4.2. Comparison with Existing Methods

To evaluate the effectiveness of the proposed method for 2D pose estimation of underwater divers, it was compared with the supervised baseline HRNet-W48 [9] and three representative semi-supervised 2D human pose estimation methods: PoseDual [12], SSPCM [11], and MultiAugs [13]. The supervised HRNet-W48 baseline used only labeled training data and served to quantify the performance gain achieved by incorporating unlabeled data through semi-supervised learning. Following the original settings of the semi-supervised methods, PoseDual, SSPCM, and MultiAugs used Joint Cutout (JC), SSCO, and JOCO + JCCM as their hard-augmentation strategies, respectively. To control for differences in backbone capacity, all methods used HRNet-W48 as the backbone of the keypoint-estimation network, together with the same data splits, pretrained initialization, basic affine augmentation, and evaluation metrics. Because the original SSPCM implementation used SimpleBaseline [16] for keypoint-heatmap estimation, its backbone was replaced with HRNet-W48 to ensure a consistent comparison, while its original pseudo-label correction mechanism and hard-augmentation strategy were retained. The proposed method used JC as the hard-augmentation strategy and is denoted by Ours (JC) in the tables.

**Quantitative comparison.** The results in Table 2 were obtained by evaluating the retained checkpoints from three training runs for each method, using random seeds 1949, 2026, and 2030, on the independent test set. Panel (a) compares body-part and overall accuracy at PCKh@0.5; panel (b) compares overall accuracy across thresholds and reports variability across runs. The proposed method achieves the highest mean overall PCKh@0.5 among all methods evaluated, at 80.07%, exceeding HRNet-W48 trained using only labeled data by 6.80 percentage points. All other semi-supervised methods also outperform the supervised baseline, indicating that, under the current data composition, incorporating 4480 unlabeled samples for semi-supervised training not only increases the amount of training data but also broadens the coverage of diverse underwater environments and appearance variations, thereby improving pose estimation performance. All semi-supervised methods use the same labeled and unlabeled training sets. Among the other semi-supervised methods, PoseDual (JC) achieves the highest mean PCKh@0.5, while the proposed method yields a further improvement of 1.61 percentage points. The proposed method also has the lowest sample standard deviation at this threshold (0.07 percentage points), indicating limited variation across the three runs. Because both PoseDual and the proposed method employ JC as the hard-augmentation strategy, this comparison controls for differences in hard augmentation. PoseDual primarily imposes consistency constraints within the single representation of keypoint heatmaps, whereas the proposed method additionally introduces cross-representation supervision between keypoint and limb representations. This result is consistent with the design motivation of using region-level limb-structure information to complement point-level keypoint supervision.

For individual body parts, Table 2a shows that the proposed method achieves the highest mean PCKh@0.5 for all seven reported body-part groups. Among these body parts, the local visual cues around the shoulder, elbow, wrist, hip, knee, and ankle can be weak under underwater degradation. The localization of these body parts is also susceptible to pose variation and occlusion. Nevertheless, the proposed method maintains relatively high localization accuracy for these parts. This result further supports the view that continuous limb-structure supervision enhances the model’s ability to represent spatial relationships among keypoints.

Table 2b further evaluates localization accuracy at stricter PCKh thresholds of 0.1, 0.2, 0.3, and 0.4. Lower thresholds require more precise keypoint localization. The proposed method achieves the highest mean scores at all four thresholds. At PCKh@0.1, it exceeds SSPCM (SSCO), the strongest comparison method at this threshold, by 0.30 percentage points. At PCKh@0.2, PCKh@0.3, and PCKh@0.4, it exceeds PoseDual (JC), the strongest comparison method at these thresholds, by 0.52, 0.75, and 1.33 percentage points, respectively. These results show that the performance advantage is maintained under stricter localization criteria.

To evaluate performance under limited annotations, 25%, 50%, and 75% subsets were selected at fixed intervals from a fixed list of labeled training samples ordered by source video and chronologically within each video. This deterministic procedure was used to spread the selected samples throughout the ordered list. The 100% setting used the full labeled training set. At each label ratio, all methods and all three runs used exactly the same labeled subset, ensuring identical labeled inputs for comparison. All semi-supervised methods used the same fixed set of 4480 unlabeled training samples under all four label settings. Table 3 reports means ± sample standard deviations across three runs with the same seeds. As shown in Table 3, at labeled-data proportions of 25%, 50%, 75%, and 100%, the proposed method achieves mean PCKh@0.5 values of 77.08%, 78.39%, 79.56%, and 80.07%, respectively, yielding the highest mean accuracy under all four settings. SSPCM (SSCO) achieves the highest mean accuracy among the comparison methods at the 25%, 50%, and 75% label ratios, whereas PoseDual (JC) is the strongest comparison method at the 100% label ratio. The corresponding improvements of the proposed method over the strongest comparison method at each ratio are 2.20, 2.04, 1.44, and 1.61 percentage points, respectively. If PoseDual (JC), the best-performing comparison method under the 100% labeled setting, is used as a common reference, the proposed method exceeds PoseDual (JC) by 2.68, 2.32, and 1.81 percentage points under the 25%, 50%, and 75% settings, respectively. In constructing supervision signals from unlabeled data, all three comparison methods rely primarily on the single representation of keypoint heatmaps. When local appearance is degraded by underwater imaging conditions, keypoint pseudo-labels are more prone to localization shifts or reduced confidence. As the proportion of labeled samples decreases, the model relies increasingly on supervision from unlabeled data, and these issues may have a more pronounced effect on training. By contrast, the proposed method introduces heatmaps that cover the limb regions between adjacent keypoints and exploits their region-level structural information through bidirectional cross-representation consistency, thereby providing complementary structural constraints when keypoint pseudo-labels are insufficiently reliable. The performance advantage of the proposed method under limited annotations is consistent with this mechanism-based analysis.

**Figure 7:**
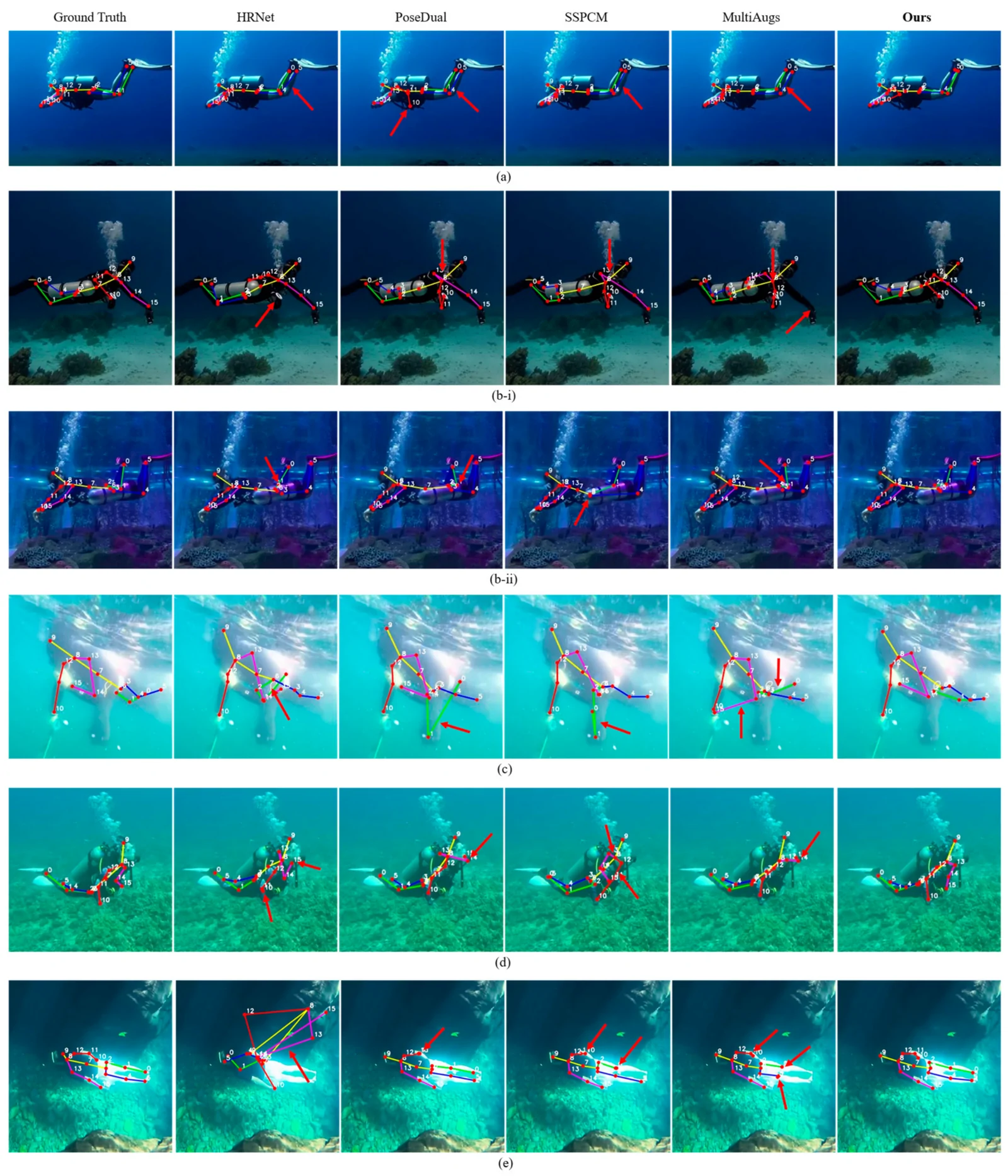
Qualitative comparisons on selected validation-set images. Columns, from left to right: Ground Truth, HRNet, PoseDual (JC), SSPCM (SSCO), MultiAugs (JOCO + JCCM), and Ours (JC). Scenes: (**a**) challenging viewpoint; (**b-i**,**b-ii**) body occlusion; (**c**) severe blur; (**d**) color cast; (**e**) strong illumination. Red arrows indicate representative keypoint-localization errors and implausible limb configurations. Colored lines indicate skeletal connections and distinguish different body parts; numbers correspond to the keypoint indices listed in Table 1.

**Qualitative comparison.** Figure 7 presents pose-estimation results from different methods on selected validation-set images from challenging underwater scenes. In the challenging-viewpoint scene, the comparison methods produce errors such as incorrect left-right assignments of the lower limbs, whereas the overall prediction of the proposed method more closely matches the ground truth. In the body-occlusion scenes, the comparison methods produce incorrect upper-limb connections and generate some anomalous lower-limb structures in the first sample. In the second sample, air-tank occlusion causes several comparison methods to mislocalize the right-knee and hip keypoints, displacing the predicted right knee–ankle connection from the actual lower leg. By contrast, the proposed method produces keypoint layouts and limb connections that more closely match the ground truth in both samples. In the severe-blur scene, all comparison methods produce implausible pose predictions, whereas the proposed method maintains a plausible overall pose. In the color-cast scene, some comparison methods mislocalize the left-wrist keypoint or predict upper-limb structures inconsistent with the actual pose, whereas the keypoint locations and connections predicted by the proposed method more closely match the ground truth. In the strong-illumination scene, strong-light interference reduces the effective visual information available from the affected limb regions, causing the comparison methods to produce predictions inconsistent with human skeletal structure; the overall pose predicted by the proposed method is closer to the ground truth. Collectively, these qualitative results illustrate closer agreement with the ground truth in the selected validation-set cases, including keypoint configurations and limb connections, consistent with the intended role of region-level limb structure in complementing point-level supervision.

The reported performance depends on the characteristics and coverage of the dataset used. Although the subsets include diverse underwater scenes and appearances and originate from non-overlapping source videos, evaluation is limited to a single dataset. Performance could decline substantially when external data differ markedly from the training data in water clarity, illumination, camera viewpoints, or diver equipment. No external-dataset evaluation has been conducted, and the magnitude of any resulting performance loss has not been quantified. Generalization beyond the evaluated conditions therefore requires further validation.

### 4.3. Ablation Study

To analyze the effects of the four consistency constraints on the keypoint and limb networks, ablation experiments were conducted using the proposed method with JC as the basis. The constraints considered were keypoint-heatmap intra-representation consistency K→K, limb-heatmap intra-representation consistency B→B, limb-to-keypoint cross-representation consistency B→K, and keypoint-to-limb cross-representation consistency K→B. All experiments used the same data splits, network initialization, hard-augmentation method, training parameters, and a fixed random seed of 1949. Six experimental configurations defined by different combinations of the four consistency constraints were evaluated and are labeled A–F in Table 4.

Table 4 reports the PCKh@0.5 and mSoftDice results for these configurations. Configuration A, which retains only the two within-network intra-representation consistency terms K→K and B→B, achieves an overall PCKh of 77.43%. The complete configuration F, which additionally includes K→B and B→K, achieves 80.13%, an improvement of 2.70 percentage points. With all other experimental settings held constant, this result indicates that adding bidirectional cross-representation supervision to within-network intra-representation consistency improves final keypoint-localization performance.

To further examine the individual and combined contributions of the two cross-representation consistency paths, configurations A, B, C, and F are compared while keeping both intra-representation consistency constraints, K→K and B→B, enabled. These four configurations cover all combinations of enabling or disabling the two cross-representation paths. Relative to configuration A, adding B→K alone yields configuration C and increases PCKh from 77.43% to 79.75%, a gain of 2.32 percentage points. By comparison, adding K→B alone yields configuration B and increases PCKh from 77.43% to 77.75%, a gain of only 0.32 percentage points. When B→K is already enabled, adding K→B increases PCKh from 79.75% in configuration C to 80.13% in configuration F, a gain of 0.38 percentage points. Thus, adding K→B provides a further gain when the reverse B→K path is present. This pattern supports the complementary roles of the two directions: K→B transfers structural targets derived from keypoint predictions to supervise the limb network, whereas B→K uses limb-network predictions to provide structural supervision to the keypoint network. Together, these paths establish bidirectional supervision between point-level keypoint and region-level limb representations.

Adding K→B alone to configuration A decreases mSoftDice from 0.7956 to 0.7851 in configuration B, showing no improvement in limb-prediction quality in this comparison. However, when the reverse B→K path is already enabled, adding K→B increases mSoftDice from 0.7926 in configuration C to 0.8028 in configuration F, while also increasing PCKh from 79.75% to 80.13%. Together with the PCKh gains observed when comparing configuration A with B and configuration C with F, these results show that, in the reported experiments, adding K→B improves limb-prediction quality when B→K is present, supporting the complementary roles of the two cross-representation paths.

The two intra-representation consistency terms also serve different roles. Removing B→B from the complete configuration F yields configuration D, in which PCKh decreases from 80.13% to 79.54% and mSoftDice decreases from 0.8028 to 0.7496. This result indicates that B→B helps maintain the quality of structural predictions produced by the limb network. Removing K→K yields configuration E, in which PCKh decreases to 79.47%, whereas mSoftDice decreases only from 0.8028 to 0.7991. This result indicates that K→K primarily acts on the keypoint network, helping to maintain keypoint-localization performance. Taken together, the two sets of results indicate that K→K and B→B help maintain the consistency of point-level keypoint and region-level limb representations, respectively, across the easy-augmentation and hard-augmentation views. The two constraints jointly provide a foundation for bidirectional cross-representation learning.

mSoftDice and PCKh do not vary in strict synchrony because they evaluate different targets. mSoftDice measures the accuracy with which the auxiliary limb network predicts the ground-truth limb structure, whereas PCKh measures the localization performance of the final keypoint network. Therefore, mSoftDice is used primarily to support the analysis of changes in limb-structure prediction quality and cannot replace PCKh as the evaluation metric for final pose-estimation performance. Although the two metrics focus on different output spaces, the complete configuration F achieves the highest PCKh and mSoftDice simultaneously, indicating that joint optimization of the four consistency constraints benefits both final keypoint localization and structural prediction by the auxiliary limb network.

## 5. Conclusions

A limb-heatmap-guided bidirectional cross-representation consistency method is proposed for semi-supervised 2D pose estimation of underwater divers. Independent keypoint and limb networks with no shared parameters are employed to learn point-level keypoint responses and region-level limb structures, respectively. For labeled data, labels for the limb network are constructed from keypoint annotations according to a predefined skeletal topology. For unlabeled data, intra-representation consistency constraints are imposed on the predictions of both networks on the easy-augmentation and hard-augmentation views. Confidence-weighted keypoint-to-limb cross-representation supervision and structure-guided limb-to-keypoint cross-representation supervision are further established. Together, these two cross-representation directions enable bidirectional information transfer between the representations, allowing continuous limb structure to provide complementary supervision for keypoint localization. Only the keypoint network is retained during inference; therefore, the auxiliary limb network introduces no additional deployment overhead.

With checkpoints selected using the validation set, quantitative results on the independent test set of the underwater diver dataset constructed for this study demonstrate that the proposed method achieves higher overall keypoint-localization accuracy than both the supervised baseline and the representative semi-supervised methods evaluated in this study. It achieves the highest mean accuracy at all evaluated label ratios. The qualitative results further show that, in the selected challenging validation-set scenes, the keypoint locations and overall pose predictions produced by the proposed method are generally closer to the ground truth. Ablation experiments support the effectiveness of both intra-representation consistency in each network and bidirectional cross-representation consistency, suggesting that point-level keypoint and region-level limb representations can provide complementary supervision for each other during learning from unlabeled data. However, these findings remain limited to a single dataset. Robustness beyond the evaluated conditions and suitability for practical deployment require further validation.

Future work will further evaluate the robustness of the proposed method under a broader range of underwater imaging conditions, including variations in water clarity, depth, and illumination, and investigate whether underwater image-formation priors or physically motivated augmentation can further improve robustness to environmental variations. Keypoint visibility and uncertainty will also be modeled in complex underwater scenes involving severe occlusion, out-of-frame limbs, and overlap among multiple divers. Confidence- or uncertainty-based weighting and filtering of limb-derived structural pseudo-labels will also be investigated to improve the reliability of supervision in the B→K path. Motion-continuity constraints between adjacent frames and cross-frame structural constraints will be introduced to further improve pose-estimation stability. In addition, dedicated field validation and reliability assessment under previously unseen conditions will be conducted before practical deployment, particularly in safety-critical scenarios. Based on this study, the research scope will be further expanded to diver behavior recognition and intention understanding, and their integration with the decision-making and control of underwater robots will be explored.

## Figures and Tables

**Table 1:** Indices and names of the diver keypoints used in this study.

Index	Keypoint Name	Index	Keypoint Name
0	Right Ankle	8	Thorax
1	Right Knee	9	Head Top
2	Right Hip	10	Right Wrist
3	Left Hip	11	Right Elbow
4	Left Knee	12	Right Shoulder
5	Left Ankle	13	Left Shoulder
6	Pelvis	14	Left Elbow
7	Spine	15	Left Wrist

**Table 2:** Quantitative comparison on the independent underwater diver test set using 100% of the labeled training data. (**a**) PCKh@0.5 by body part and overall (mean, %). (**b**) Overall PCKh at different thresholds (mean ± SD, %).

(**a**)								
**Method** **(Hard Augmentation)**	**Head**	**Shoulder**	**Elbow**	**Wrist**	**Hip**	**Knee**	**Ankle**	**Overall**
HRNet-W48 (Sup.) [9]	90.91	76.86	70.99	70.12	70.67	67.41	67.11	73.27
PoseDual (JC) [12]	92.72	81.04	75.28	74.26	76.11	73.47	76.21	78.45
SSPCM (SSCO) [11] †	92.96	79.58	74.50	75.02	76.11	73.90	74.69	78.01
MultiAugs (JOCO + JCCM) [13]	92.77	78.49	72.93	73.24	74.65	73.68	74.96	77.33
Ours (JC)	**93.06**	**82.90**	**77.46**	**76.26**	**78.37**	**74.97**	**77.24**	**80.07**
(**b**)								
**Method** **(Hard Augmentation)**	**PCKh@0.1**		**PCKh@0.2**	**PCKh@0.3**		**PCKh@0.4**	**PCKh@0.5**	
HRNet-W48 (Sup.) [9]	20.17 ± 0.23		41.67 ± 0.38	57.78 ± 0.35		67.33 ± 0.12	73.27 ± 0.23	
PoseDual (JC) [12]	22.77 ± 0.09		46.69 ± 0.24	64.07 ± 0.10		73.14 ± 0.19	78.45 ± 0.17	
SSPCM (SSCO) [11] †	22.79 ± 0.43		45.80 ± 0.35	62.57 ± 0.43		72.58 ± 0.23	78.01 ± 0.44	
MultiAugs (JOCO + JCCM) [13]	22.03 ± 0.47		45.45 ± 0.54	62.21 ± 0.83		71.86 ± 0.91	77.33 ± 0.94	
Ours (JC)	**23.09 ± 0.36**		**47.22 ± 0.53**	**64.82 ± 0.40**		**74.47 ± 0.17**	**80.07 ± 0.07**	

**Note:** † SSPCM uses HRNet-W48 in place of SimpleBaseline for its keypoint network. Bold and underlined values indicate the highest and second-highest means in each column, respectively. SD denotes sample standard deviation.

**Table 3:** Comparison of overall PCKh@0.5 (%) at different proportions of labeled training data.

Method (Hard Augmentation)	Proportion of Labeled Training Data			
	25%	50%	75%	100%
PoseDual (JC) [12]	74.40 ± 0.57	76.07 ± 0.82	77.75 ± 0.62	78.45 ± 0.17
SSPCM (SSCO) [11] †	74.89 ± 1.26	76.35 ± 0.58	78.12 ± 0.69	78.01 ± 0.44
MultiAugs (JOCO + JCCM) [13]	74.02 ± 0.25	75.13 ± 0.33	76.31 ± 0.59	77.33 ± 0.94
Ours (JC)	**77.08 ± 0.29**	**78.39 ± 0.30**	**79.56 ± 0.42**	**80.07 ± 0.07**

**Note:** Values are reported as mean ± sample standard deviation. † SSPCM uses HRNet-W48 in place of SimpleBaseline for its keypoint network. Bold and underlined values indicate the highest and second-highest means in each column, respectively.

**Table 4:** Ablation results for six configurations defined by different combinations of the four consistency constraints.

Configuration	K→K	B→B	B→K	K→B	PCKh@0.5	mSoftDice
A	✓	✓	—	—	77.43	0.7956
B	✓	✓	—	✓	77.75	0.7851
C	✓	✓	✓	—	79.75	0.7926
D	✓	—	✓	✓	79.54	0.7496
E	—	✓	✓	✓	79.47	0.7991
F	✓	✓	✓	✓	80.13	0.8028

**Note:** A check mark indicates that the corresponding constraint is included, whereas a dash indicates that it is excluded.

## Data Availability

The dataset is not publicly available due to privacy concerns involving individuals in the online source videos. Data access is subject to privacy restrictions and may be requested from the corresponding author. The training code, configurations, and evaluation scripts are publicly available at https://github.com/one-core-half-threads/LimbBCRC-DiverPose (accessed on 26 October 2026).
